# Comparing the Attitudes of Healthcare Professionals and Cancer Patients Toward the Integration and Perceived Effectiveness of Complementary and Alternative Medicine

**DOI:** 10.3390/healthcare13212818

**Published:** 2025-11-06

**Authors:** Ljerka Armano, Martina Trnčević, Andrea Armano, Aneta Perak, Aleksandar Racz

**Affiliations:** 1 UHC Sestre Milosrdnice, 10000 Zagreb, Croatia; ljerka.armano@kbcsm.hr; 2 Faculty of Medicine, University of Rijeka, 51000 Rijeka, Croatia; martina.trncevic@yahoo.com; 3 Aspira University of Applied Sciences, 21000 Split, Croatia; andrea.armano@aspira.hr; 4 Faculty of Teacher Education, University of Zagreb, 10000 Zagreb, Croatia; perakaneta@gmail.com

**Keywords:** health attitudes, complementary and alternative medicine, integrative medicine, cancer patients, nursing health personnel

## Abstract

**Background:** Complementary and alternative medicine (CAM) is increasingly used to supplement evidence-based medicine (EBM), especially in the treatment of cancer patients. **Objective:** The study aimed to analyze the beliefs, attitudes, and expectations of healthcare professionals and cancer patients regarding the integration of CAM into the Western medical system, and to examine differences between these groups in their expectations for CAM effectiveness. The hypotheses were that there are no statistically significant differences in attitudes between healthcare professionals (nurses and physicians) and cancer patients regarding CAM integration into EBM and the effectiveness of CAM. **Methods:** The study was conducted on a stratified sample of 832 respondents: 411 cancer patients and 421 health professionals (100 physicians and 321 nurses). Validated questionnaires based on CHBQ and IMAQ instruments were used. Descriptive and inferential statistics were applied in the analysis. **Results:** Patients showed a significantly more positive attitude toward CAM methods than healthcare professionals. A total of 70% of respondents believed that CAM should be integrated into EBM. Most respondents supported formally noting CAM therapies in medical records and including them in medical history. Healthcare professionals, especially physicians, expressed greater concerns about the effectiveness of CAM, while patients had more positive expectations about its benefits. **Conclusions:** The findings suggest that healthcare professionals require better education on CAM therapies and that greater openness is necessary to integrate these methods into medical practice. Although patients have high expectations for CAM, its inclusion in the formal medical system requires further research on safety and efficacy.

## 1. Introduction

The US National Institutes of Health (NIH) defines complementary and alternative medicine (CAM) as therapies that are not part of standard medical practice [1]. The World Health Organization (WHO) further explains that CAM refers to treatments that are not fully integrated into the mainstream healthcare system [2]. The distinction between evidence-based medicine (EBM) and CAM remains fluid, as some methods, such as acupuncture, are gradually being incorporated into clinical settings, while others remain peripheral due to insufficient scientific evidence. Medicine, as a field grounded in both science and experience, is continuously evolving to include patient-oriented and holistic perspectives [3].

CAM approaches are increasingly recognized for their holistic nature, which considers health in physical, psychological, emotional, and spiritual dimensions. This integrative focus enhances patient satisfaction and promotes treatment adherence through empowerment and active participation in recovery [4,5]. At the same time, the growing use of CAM raises important concerns. Insufficient scientific validation, a lack of standardization, and weak regulatory frameworks result in variations in quality and safety [6,7,8]. Risks of side effects or harmful interactions with conventional oncology therapies persist, especially when communication between patients and healthcare providers is inadequate or hindered by fear of judgment or insufficient professional knowledge [6].

In oncology, shared decision-making (SDM) is crucial when patients consider CAM options alongside conventional treatments. Many patients seek complementary therapies to relieve symptoms, reduce stress, and improve quality of life [9,10,11,12,13,14,15,16,17,18]. Evidence supports specific CAM methods, such as acupuncture, massage, and hypnosis, in reducing pain and procedural anxiety, while yoga, mindfulness, and music therapy show positive, though sometimes less conclusive, outcomes [19,20,21]. These interventions increasingly complement conventional cancer treatments, reflecting a shift toward person-centered and integrative care models. 

The prevalence of CAM use among cancer patients varies globally. A nationwide survey in the United States reported that 33.3% of patients and survivors used CAM therapies within the previous year, most commonly herbal supplements (35.8%), chiropractic manipulation (25.4%), massage (14.1%), and meditation-based practices (6.9%) [22]. Across Europe, approximately 39.1% of cancer patients use CAM, with rates influenced by cancer type and sociodemographic factors [23]. In Croatia, reported CAM use reaches 55.6% among cancer patients and 32.2% among healthcare professionals [24]. These figures confirm that CAM represents a significant aspect of supportive cancer care, justifying its examination within the context of this study.

Cross-cultural studies show that CAM use reflects complex psychosocial, cultural, and experiential factors. Previous study [25] reported that 40% of European cancer patients use at least one form of CAM, primarily herbal medicine, acupuncture, or massage. In Asia, Kwon et al. [26] found similar trends, while Schils et al. [27] confirmed positive attitudes toward natural therapies in Belgium. Meta-analyses indicate that acupuncture, mindfulness, and yoga can alleviate pain, fatigue, and anxiety in cancer patients [28,29,30], and recent studies suggest that the COVID-19 pandemic further increased CAM use as a coping strategy [31,32,33,34,35]. Parallel to patient practices, healthcare professionals acknowledge the potential of CAM but emphasize the need for regulation, education, and quality control [36]. Consequently, this study aimed to analyze and compare the beliefs and attitudes of healthcare professionals and cancer patients regarding the integration of CAM into evidence-based medicine and to examine differences in their perceptions of its effectiveness. Two null hypotheses were formulated: (H_01_) there are no statistically significant differences between healthcare professionals (nurses and physicians) and cancer patients regarding the integration of CAM into EBM, and (H_02_) there are no statistically significant differences between these groups regarding their perceptions of CAM effectiveness.

## 2. Materials and Methods

### 2.1. Participants

The cross-sectional study was conducted from November 2022 to May 2023 at the Sisters of Mercy University Hospital Centre (UHC Sisters of Mercy) in Zagreb, Croatia. The sample consisted of 1200 participants, comprising approximately 400 cancer patients and 800 healthcare professionals. Stratified random sampling was used to ensure proportional representation of physicians and nurses/technicians (in the Croatian healthcare system, “technicians” refers to nurses) within the healthcare professional group. The term “medical technician” traditionally denotes a male nurse, while “nurse” refers to a female professional. Both titles describe the same professional category with identical education and competencies. For clarity and consistency with international usage, this study uses the term “nurse” inclusively to refer to all nursing professionals, regardless of gender or national title. A total of 832 participants were included, comprising 411 cancer patients and 421 healthcare professionals, consisting of 100 physicians and 321 nurses. Eligible participants were approached during their hospital visits or work shifts. Trained researchers recruited cancer patients during outpatient or inpatient visits, while healthcare professionals were recruited through departmental meetings and workplace announcements. All participants provided written informed consent before participating.

The estimated sample size was calculated based on the proportional representation of healthcare professionals and newly registered cancer patients in the study area. The aim was to include about 30% of newly registered cancer patients over a 6-month period. Healthcare professionals took part in the survey to reflect the distribution of the workforce across oncology departments. The final participation rates and a detailed demographic breakdown of the sample are shown in the Section 3.

Participants were chosen using a proportionally stratified random sample to ensure all key groups were represented. The study included two main strata: Stratum 1, consisting of cancer patients diagnosed with cancer according to the International Classification of Diseases, regardless of disease stage; and Stratum 2, comprising healthcare professionals involved in oncology care. Participants in the second stratum were further divided into two subgroups: Stratum 2.1 (physicians) and Stratum 2.2 (nurses).

Cancer patients were recruited in outpatient clinics and inpatient oncology wards by trained researchers who provided verbal and written information about the study. Eligible participants were adults (≥18 years) with a confirmed cancer diagnosis, receiving or having recently completed oncology treatment, and capable of giving informed consent. Participation was voluntary and anonymous. After providing written consent, patients completed the questionnaire independently or with minimal assistance when required due to health limitations. Patients were systematically recruited from newly registered oncology cases over six months to minimize selection bias.

Healthcare professionals were recruited from oncology and related departments (hematology, surgery, gynecology, otolaryngology, internal medicine) using a proportionally stratified random sampling method. Physicians and nurses were informed about the study via departmental announcements, hospital e-mails, and brief presentations at staff meetings. Participation was voluntary and took place outside working hours to avoid any workplace influence.

To ensure representativeness, the sample size was calculated proportionally based on the number of active healthcare staff and newly registered cancer patients during the study period. This stratified approach ensured a balanced representation of both patients’ and professionals’ perspectives. All participants were informed that data collection would be anonymous, that no identifying information would be recorded, and that they could withdraw at any time without consequences. This means that informed consent was obtained from all participants prior to their participation in the study. Participants were briefed on the study’s purpose, their voluntary participation, and the confidentiality of their responses. CAM was defined according to NIH and WHO standards, and examples were provided to ensure a shared understanding among participants.

Notes on terminology: In this study, the term ‘nurse’ is used generically to refer to nurses and other health professionals, regardless of gender, professional standards, or language.

### 2.2. Questionnaire and Data Collection

Two structured questionnaires were used: one for healthcare professionals (physicians and nurses) and one for cancer patients. Both were developed from the same conceptual framework, comprising parallel sets of questions regarding the perceived effectiveness, safety, and integration of CAM. The patient version included 24 items, and the professional version included 26 items (the additional two items addressed professional responsibilities and communication with patients). The wording of items was adapted to the respondent’s perspective (“How do you perceive…?” for patients; “How do your patients perceive…?” for professionals). Despite these minor linguistic adjustments, the questionnaires shared identical thematic domains and Likert-scale structures, allowing for direct comparison across the three analytical groups: physicians, nurses, and cancer patients.

To emphasize, both questionnaires were adapted, with only minor modifications in the socio-demographic section, from previously validated instruments: the CAM Health Belief Questionnaire (CHBQ) and the Integrative Medicine Attitude Questionnaire (IMAQ) [37,38]. The detailed structure, validation process, and psychometric properties of both instruments have already been thoroughly presented in a previous paper (24).

Eligible participants were invited to take part during their hospital visits or work shifts. Trained researchers recruited cancer patients during outpatient appointments or inpatient stays, while healthcare professionals were approached through departmental meetings and workplace announcements. All respondents provided written informed consent before participation.

To reduce non-response and improve clarity, the surveys were conducted face-to-face with a trained interviewer assisting participants in completing the questionnaires. This approach ensures consistent, accurate responses.

The data was anonymized and securely stored in a protected database, with trained staff responsible for data entry and management. Responses were carefully checked for accuracy and completeness to ensure data integrity. Additionally, all identifying information was removed to protect participants’ confidentiality.

### 2.3. Statistical Analysis

The collected data were organized according to the research objectives and presented in both text and table formats to clearly illustrate attitudes, preferences, and behavioral tendencies related to CAM. Both descriptive and inferential statistical methods were employed. Descriptive statistics summarized the data using raw figures, such as frequencies, percentages, and measures of central tendency, including means, standard deviations, and ranges (minimum and maximum). The means of three or more groups were compared using a one-way analysis of variance (ANOVA), assuming the data followed a normal distribution. If the ANOVA results indicated statistically significant differences, a post hoc Tukey test was performed to identify which groups differed significantly. This approach facilitated a more detailed analysis of attitudes and experiences regarding CAM.

## 3. Results

A total of 421 healthcare professionals participated in the study, including 100 Physicians and 321 nurses. Results are presented for three groups—physicians, nurses, and cancer patients—based on parallel items drawn from the two harmonized questionnaires described above. The sample had a higher proportion of female participants (70.6%), reflecting the trend toward feminization in healthcare professions. Among nurses, 88.2% were women, compared with 68% of physicians. Most patients were also female (57.4%), although the gender ratio was more balanced with 42.6% male patients. The participants’ ages varied, with the highest concentrations in the 41–50 (24.8%) and 51–60 (25.2%) age groups. Notably, no physicians were over 60 years old, whereas 44.3% of patients fell into this age group. The largest age groups among nurses were those aged 20–30 years (26.5%) and 41–50 years (26.2%). This distribution may reflect job responsibilities, workload, and demographic trends in cancer patients. Unsurprisingly, all the physicians held a university degree. Among nurses, 34.6% held a university degree, and 22.2% held an associate’s degree or a similar qualification in a health-related field, likely due to the complexity of oncology care. Regarding marital status, 70.3% of participants were married or in a relationship, with the highest percentages among physicians (72%) and patients (73.2%). Most participants (72.1%) lived in urban areas, which aligns with the availability of healthcare facilities in Croatia. A total of 81.5% of respondents reported a religious affiliation, with the highest prevalence among nurses (85%) and physicians (84%). Among patients, 78.1% identified themselves as spiritual, while atheism was reported by 4.3% across all groups. None of the physicians had more than 35 years of professional experience, possibly due to managerial roles and a heavy workload. Most were between 16 and 25 years old (34%). The nurses had a more balanced distribution, with the largest groups comprising those with 16–25 (24.9%) and 26–35 (24.3%) years of experience, indicating stability in the profession. A significant proportion of physicians (56%) were directly involved in oncology care, compared with 22.9% of nurses; the remainder worked in related departments. An income analysis revealed inequalities: 97% of physicians reported earning more than the national average, compared with 26.5% of nurses. Of patients, 50.4% had incomes below the average, likely reflecting the financial impact of their illness. This section of the questionnaire focuses on analyzing how CAM is integrated with EBM and explores respondents’ beliefs and attitudes about the appropriate extent to which CAM should be incorporated into EBM. For transparency and ease of interpretation, the main analytical tables are presented in the manuscript. In contrast, descriptive data on the level of agreement with each statement are presented in the Supplementary Files. Table 1 presents the mean and standard deviation of respondents’ beliefs and attitudes regarding the integration of CAM into EBM, and Table 2 displays the statistical significance of the differences in respondents’ beliefs and attitudes based on Tukey’s test.

Patients have the most positive attitudes toward CAM, while nurses tend to fall in the middle, leaning more toward patients than toward doctors. Overall, physicians expressed a more cautious and moderate level of support, with greater concern about integrating CAM methods into institutions. The highest level of consensus was reached across all groups regarding the importance of communication. Participants agreed that patients should inform their physicians of their use of CAM and consult a qualified professional before its application. Notably, patients strongly support integrating the “best” elements from both conventional and complementary systems, showing a desire for a holistic approach to treatment. At the same time, healthcare professionals expressed clear resistance to the financial integration of CAM, especially regarding reimbursement by the Croatian Health Insurance Fund. Negative views of CAM as dangerous were rarely voiced, especially among patients and nurses. Support for including CAM in patient history and medical records further reinforces the view that integration is advantageous. However, it requires a structured approach and extra professional training.

The post hoc analysis (Tukey’s test) revealed statistically significant differences in attitudes among physicians and the other two groups—patients and nurses—regarding nearly all examined statements. Physicians consistently showed lower agreement with statements supporting the integration of complementary and alternative medicine (CAM) into conventional medical practice, indicating a professional distance and a higher threshold for accepting CAM methods. In contrast, patients and nurses were more receptive to incorporating CAM into various healthcare areas. Interestingly, most statements showed no significant differences between nurses and patients, which may indicate a shared perspective and increased openness toward practices that go beyond traditional biomedical frameworks. The comparative data show that nurses occupy an intermediate position between the cautious skepticism of physicians and the experiential openness of cancer patients. Their mean scores on most CAM-related attitude items were higher than those of physicians but lower than those of patients, reflecting a balanced perspective that combines professional prudence with empathy toward patients’ holistic preferences. This empirically observed pattern supports the interpretation of nurses as intermediaries within the healthcare team, bridging clinical caution with patient-centered understanding. One notable exception, where no significant differences were found among the groups, involved the statement about physicians’ perceived resistance to using CAM. Conversely, significant differences in attitudes toward funding, access, and education related to CAM highlight clear disagreements and emphasize where institutional change is most needed for the successful integration of CAM.

The data from Table 3 show the average values and standard deviations of respondents’ beliefs and attitudes about the effectiveness of CAM. Patients and nurses are significantly more likely to accept the core beliefs of the philosophy behind complementary and alternative medicine (CAM).

The analysis of attitudes toward the effectiveness of complementary and alternative medicine (CAM) shows that patients are more receptive to the core principles behind many CAM approaches. Meanwhile, healthcare professionals tend to have more reservations, especially regarding statements based on holistic, vitalistic, or spiritual principles.

Patients generally trust concepts such as inner energy, the body’s natural healing ability, and the importance of balancing life forces as vital to their health. They also prefer incorporating patients’ beliefs and values into healthcare, favoring personalized, experiential treatment methods. On the other hand, healthcare professionals tend to be more cautious about claims that contradict scientifically proven methods. This is especially clear in their reactions to statements involving divine healing powers, traditional folk wisdom, or the idea that therapies without scientific support should be banned. However, it’s important to note that outright rejection of CAM is uncommon among healthcare providers. Instead, their responses often lean toward neutrality or slight agreement, reflecting a nuanced rather than an absolute stance. The view that CAM includes approaches that could also benefit conventional medicine has moderate support from both groups, suggesting potential for dialogue and mutual enrichment. The most notable difference is in emotional reactions, such as excitement about CAM or anger toward those who believe in its effectiveness, where patients display noticeably stronger emotional engagement. Among healthcare professionals, especially physicians, the strongest support is for statements emphasizing the importance of incorporating patients’ values and beliefs into the treatment process, highlighting a focus on personalized care. However, the same respondents show low agreement with claims about self-healing, alternative sources of knowledge such as folk medicine, and the healing power of a higher power. This pattern reflects a strong alignment of medical professional identity with the scientific medicine paradigm. In contrast, nurses and patients are more likely to agree on concepts such as inner energy, balancing life forces, and holistic views of health. However, even within these groups, there is no evidence of uncritical idealization. Interestingly, statements made in reverse—such as describing CAM as a threat to public health or simply a placebo—received the highest average ratings from patients, likely due to confusion or mixed feelings about CAM’s effectiveness, which reflects both acceptance of its potential and awareness of ongoing debates. Perceptions of CAM’s effectiveness vary by professional role and exposure to alternative approaches. Physicians generally adopt a rational, skeptical viewpoint, while patients and nurses tend to support principles aligned with holistic and experiential care models.

The results of the Tukey post hoc test presented in Table 4 confirm significant differences in beliefs about the effectiveness of complementary and alternative medicine (CAM) among physicians, nurses, and patients. The most notable differences are between physicians and the other two groups, with physicians being statistically more likely to reject statements reflecting a holistic, vitalistic, or spiritual view of health. Patients and nurses, on the other hand, show far fewer differences—many of the statements reveal no statistically significant distinction between these two groups. This reinforces the similarity of their perspectives on concepts such as inner energy, traditional knowledge, and the potential of CAM therapies. The main differences between physicians and other groups relate to beliefs about trust in CAM healing sources, such as the body’s self-healing ability, the potential benefits of complementary and alternative medicine (CAM) alongside conventional medicine, and belief in a higher healing power.

In summary, the results demonstrate clear and statistically significant differences in attitudes toward complementary and alternative medicine (CAM) among physicians, nurses, and cancer patients. Physicians expressed the greatest caution and skepticism, primarily emphasizing scientific evidence and patient safety. At the same time, nurses displayed more integrative and balanced perspectives, reflecting their dual orientation toward evidence-based care and patient-centered understanding. Cancer patients showed the most positive attitudes toward CAM use and the strongest belief in its perceived benefits. Across all groups, there was broad support for integrating CAM into evidence-based medical practice, provided that such integration is guided by professional education, quality assurance, and transparent documentation within the healthcare system.

## 4. Discussion

The present study explored differences in attitudes toward complementary and alternative medicine (CAM) among physicians, nurses, and cancer patients, focusing on perceived effectiveness, safety, and the role of CAM within evidence-based medicine. The findings revealed a clear and statistically significant gradient of attitudes across the three groups. Physicians expressed the most cautious and skeptical perspectives, prioritizing scientific evidence, patient safety, and regulatory control. Nurses demonstrated a balanced and integrative approach, emphasizing empathy and the inclusion of patients’ holistic needs in care. Cancer patients showed the highest level of acceptance and trust in CAM, driven by experiential and emotional dimensions of healing. These patterns suggest that attitudes toward CAM are not merely based on knowledge or exposure but are deeply rooted in professional identity, ethical orientation, and the nature of interaction with patients.

Differences between physicians, nurses, and patients highlight the importance of professional role and epistemological orientation. Physicians’ skepticism reflects their responsibility for clinical decisions and adherence to scientifically validated practice, while nurses’ openness reflects their relational proximity and communication-based role in patient care. The position of nurses between biomedical caution and patient-centered openness allows them to act as mediators in the process of CAM integration. This bridging role may be critical for reducing communication barriers and promoting patient safety through appropriate documentation and consultation. Across all groups, participants emphasized the need for structured, evidence-informed integration of CAM into the healthcare system, including formal education and standardized recording of CAM use in patient histories.

Our results align with previous international research showing similar professional differences. Bjerså et al. [39] reported that these variations often arise from distinct clinical responsibilities and patient contact intensity. Studies also confirm that nurses tend to show more positive attitudes toward CAM than physicians [40,41,42,43,44,45,46,47,48,49], largely because of their holistic care philosophy and the overlap between nursing values and CAM principles [50]. In the Netherlands, 37% of nurses were familiar with integrative medicine, and 83% regarded it as an important healthcare innovation [51]. Our findings confirm this orientation, as over 70% of respondents supported documenting CAM use in patient records, consistent with previous calls for greater transparency and monitoring of natural products in oncology [52,53,54,55]. This widespread acceptance underscores the practical need for multidisciplinary dialogue and regulatory frameworks that ensure safety while respecting patient autonomy.

In contrast, some studies found divergent patterns. Furlow et al. [56] observed that physicians in the United States often hold more positive views of CAM than their patients, possibly reflecting differences in healthcare education and institutional policies. In Croatia, Radovčić and Nola [57] found that 88% of general practitioners supported integrating CAM into evidence-based medicine, but that strong resistance persisted among hospital physicians. Jurković et al. [58] and later surveys of nursing and physiotherapy students [59] also demonstrated a consistently positive trend among younger professionals, suggesting that generational change may support greater acceptance of integrative approaches. Our study, based on a larger and more heterogeneous sample, confirms these findings and emphasizes the necessity of introducing CAM content into undergraduate and continuing medical education to harmonize attitudes across professions.

Cultural and psychosocial factors also influence how CAM is perceived and practiced. Belief in vital energy and the body’s innate healing ability remains a core feature of CAM philosophy and is frequently associated with spirituality [60,61,62,63,64,65,66]. The concept of life force or universal energy reflects a worldview in which health results from the balance between internal and external forces. In this context, illness is understood not only as biological dysfunction but also as a disruption of harmony between mind, body, and environment. Such views, sometimes linked to New Age and Eastern traditions [67], are more common among nurses and patients, who tend to interpret healing through experiential and existential dimensions. Physicians, on the other hand, are more likely to rely on empirical verification, which explains their lower agreement with statements referring to divine or energetic sources of healing. These cultural dimensions must be recognized when developing communication strategies for patient counseling.

From a policy and practice perspective, the study highlights several key implications. The high degree of patient openness to CAM underscores the need for healthcare institutions to implement clear procedures for documenting CAM use and educating professionals about potential interactions. Integrating CAM into clinical documentation, as supported by over two-thirds of respondents, is crucial for patient safety and coordination of care. At the same time, the results point to the need for interdisciplinary education on the evidence base, safety, and ethical aspects of CAM. Regulatory bodies should encourage certification standards and cross-sector collaboration between conventional and complementary practitioners to ensure quality, accountability, and equitable access.

The study has several limitations. First, the sample was collected at a single hospital, which may limit the generalizability of the results to the broader cancer patient and healthcare professional population in Croatia. The fact that only patients from a single hospital and healthcare professionals from specific departments were included may have led to an imbalance in the representation of different professional and patient perspectives. In addition, there is a possibility that socially desirable responses were given, especially given the sensitivity of the topic of CAM, which could influence the expression of actual attitudes and practices. The study is cross-sectional, meaning that it captures attitudes and beliefs simultaneously. A longitudinal approach would be necessary to track changes over time. The lack of current epidemiological data and the impact of the COVID-19 pandemic may also have influenced respondents’ perceptions and answers. In contrast, some respondents may have been insufficiently informed about CAM, which could have affected the accuracy of responses. Methodological limitations include selection bias and the possibility of recall error, particularly among cancer patients and healthcare professionals who may have selectively interpreted their experiences. The study relies on questionnaires, which may lead to response bias as participants may have given socially desirable answers or misunderstood some survey items. Finally, the complexity of the questionnaire may have made it difficult for older respondents and those with lower levels of education to participate. At the same time, some answers may have been influenced by professional norms and institutional expectations. In addition, the study relied exclusively on quantitative, self-reported data, which restricts the depth of insight into personal experiences and reasoning behind participants’ attitudes toward CAM.

Future studies should therefore adopt a mixed-method approach, combining quantitative surveys with qualitative interviews or focus groups, to explore the underlying motivations, beliefs, and contextual factors that shape perceptions of CAM among healthcare professionals and cancer patients. Comparative, multi-centre, and longitudinal designs are also recommended to enhance external validity and provide a more comprehensive understanding of attitudes toward integrative medicine across healthcare settings. From a public health and policy perspective, these findings suggest that the successful integration of CAM requires not only clinical validation but also a value-based framework that respects professional diversity and patient autonomy. Recognizing and addressing these attitudinal differences can help design targeted educational and communication strategies that foster collaboration, reduce skepticism, and promote safe, patient-centered integrative care. At the same time, further research is warranted to explore how CAM can be effectively and safely provided within the healthcare system—including models of organization, professional training, and regulatory mechanisms that ensure both accessibility and quality of care.

## 5. Conclusions

The conclusions of this study summarize the results and focus on testing the hypotheses. Based on the findings, we offer recommendations for future research and practical guidelines to improve the understanding and integration of CAM into clinical practice and medical education. The first hypothesis, which suggested that there are no significant differences in beliefs and attitudes between healthcare professionals and cancer patients regarding the integration of CAM into conventional medicine, was rejected. Significant differences were identified among physicians, patients, and nurses regarding their attitudes toward integrating CAM. Conversely, the differences between nurses and patients were less noticeable. The second hypothesis, which stated that there are no significant differences in beliefs and attitudes regarding the effectiveness of individual CAM methods, was also rejected. The results showed that patients had more positive expectations and greater confidence in CAM therapies than healthcare professionals, with physicians expressing the most skepticism. At the same time, nurses were more open to the potential benefits of CAM. Overall, the research revealed a clear gap in how patients and healthcare professionals perceive and expect complementary and alternative medicine (CAM), with patients showing greater support for its inclusion. Most respondents favored officially recording CAM use in medical records, indicating a growing acceptance of its potential role in healthcare. Physicians were more skeptical, while nurses were more open to using it. These differences may stem from variations in training, job responsibilities, and access to healthcare resources.

## Figures and Tables

**Table 1:** Mean values and standard deviation of respondents’ beliefs and attitudes toward the integration of CAM into EBM.

Statement	Physicians		Nurses		Patients	
	M	SD	M	SD	M	SD
Although they don’t talk about it. Patients utilise some therapies from complementary and alternative medicine (CAM).	2.99	1.087	3.67	0.983	3.74	0.958
It’s a shame that CAM methods are not talked about enough and are rarely used.	2.23	1.153	3.68	1.113	3.77	1.026
It is positive that there are CAM treatment methods.	2.68	1.072	3.78	0.977	3.83	0.932
CAM should be integrated with the methods of EBM and conventional medicine.	2.54	1.019	3.70	1.074	3.93	0.897
CAM therapies encompass ideas and methods that, when integrated into the system of EBM medicine, can benefit everyone.	2.46	1.150	3.71	1.022	4.08	0.870
Clinical medicine should integrate the best of CAM and EBM medicine.	2.68	1.246	4.00	0.978	4.20	0.893
There is strong resistance among physicians to using CAM in patients involved in diagnostic and therapeutic processes.	3.36	0.798	3.33	0.937	3.37	0.902
I believe that CAM methods are dangerous and harmful to health.	3.44	0.946	2.07	0.995	1.89	0.834
CAM treatment should be fully covered by the Croatian Health Insurance Fund (HZZO).	1.90	1.078	3.38	1.098	3.66	0.934
CAM therapies should be available to patients at the primary care level.	2.22	1.177	3.53	1.084	3.87	0.952
A record of the application of CAM should be kept in the patient’s medical record/documentation.	3.44	1.095	4.07	1.005	4.02	0.885
When taking an anamnesis. anamnestic data on CAM applications should be taken.	3.50	1.096	4.12	1.017	4.04	0.824
The patient must inform their medical team about the use of CAM.	3.83	0.965	4.22	0.957	4.17	0.850
Patients should consult their physicians or therapist before using CAM.	3.66	0.966	4.17	0.949	4.28	0.808
Healthcare professionals should be trained to talk to patients about the most commonly used CAM methods.	2.69	1.098	3.95	1.030	4.19	0.814

M (arithmetic mean); SD (standard deviation).

**Table 2:** Statistical significance of differences in beliefs and attitudes among respondents regarding integrating CAM into EBM: Tukey’s test results.

Statement	(i) By Profession/ Status	(j) By Profession/ Status	Mean Value (ij)	Standard Error	*p* *	95% Confidence Interval	
						Lower Bound	Lower Bound
Although they don’t talk about it. Patients actually use some of the therapies from the field of CAM.	Physician	Nurse	−0.680 *	0.113	0.000	−0.94	−0.42
		Patient	−0.755 *	0.110	0.000	−1.01	−0.50
	Nurse	Physician	0.680 *	0.113	0.000	0.42	0.94
		Patient	−0.075	0.073	0.564	−0.25	0.10
	Patient	Physician	0.755 *	0.110	0.000	0.50	1.01
		Nurse	0.075	0.073	0.564	−0.10	0.25
It is positive that there are CAM treatment methods.	Physician	Nurse	−1.099 *	0.111	0.000	−1.36	−0.84
		Patient	−1.150 *	0.108	0.000	−1.40	−0.90
	Nurse	Physician	1.099 *	0.111	0.000	0.84	1.36
		Patient	−0.051	0.072	0.760	−0.22	0.12
	Patient	Physician	1.150 *	0.108	0.000	0.90	1.40
		Nurse	0.051	0.072	0.760	−0.12	0.22
There is strong resistance among physicians to the use of CAM in patients involved in diagnostic and therapeutic processes.	Physician	Nurse	0.030	0.104	0.955	−0.21	0.27
		Patient	−0.010	0.101	0.995	−0.25	0.23
	Nurse	Physician	−0.030	0.104	0.955	−0.27	0.21
		Patient	−0.040	0.067	0.827	−0.20	0.12
	Patient	Physician	0.010	0.101	0.995	−0.23	0.25
		Nurse	0.040	0.067	0.827	−0.12	0.20
A record of the use of CAM should be kept in the patient’s medical record/documentation.	Physician	Nurse	−0.632 *	0.110	0.000	−0.89	−0.37
		Patient	−0.582 *	0.107	0.000	−0.83	−0.33
	Nurse	Physician	0.632 *	0.110	0.000	0.37	0.89
		Patient	0.050	0.071	0.766	−0.12	0.22
	Patient	Physician	0.582 *	0.107	0.000	0.33	0.83
		Nurse	−0.050	0.071	0.766	−0.22	0.12
When taking an anamnestic data, CAM applications should be taken into consideration.	Physician	Nurse	−0.615 *	0.107	0.000	−0.87	−0.36
		Patient	−0.541 *	0.104	0.000	−0.79	−0.30
	Nurse	Physician	0.615 *	0.107	0.000	0.36	0.87
		Patient	0.074	0.070	0.540	−0.09	0.24
	Patient	Physician	0.541 *	0.104	0.000	0.30	0.79
		Nurse	−0.074	0.070	0.540	−0.24	0.09
CAM should be integrated with EBM methods. Official medicine.	Physician	Nurse	−1.161 *	0.113	0.000	−1.43	−0.90
		Patient	−1.392 *	0.110	0.000	−1.65	−1.13
	Nurse	Physician	1.161 *	0.113	0.000	0.90	1.43
		Patient	−0.231 *	0.073	0.005	−0.40	−0.06
	Patient	Physician	1.392 *	0.110	0.000	1.13	1.65
		Nurse	0.231 *	0.073	0.005	0.06	0.40
The patient must inform their medical team about the use of CAM.	Physician	Nurse	−0.391 *	0.104	0.001	−0.63	−0.15
		Patient	−0.340 *	0.101	0.002	−0.58	−0.10
	Nurse	Physician	0.391 *	0.104	0.001	0.15	0.63
		Patient	0.051	0.068	0.732	−0.11	0.21
	Patient	Physician	0.340 *	0.101	0.002	0.10	0.58
		Nurse	−0.051	0.068	0.732	−0.21	0.11
CAM treatment should be fully covered by the Croatian Health Insurance Fund (HZZO).	Physician	Nurse	−1.483 *	0.117	0.000	−1.76	−1.21
		Patient	−1.762 *	0.113	0.000	−2.03	−1.50
	Nurse	Physician	1.483 *	0.117	0.000	1.21	1.76
		Patient	−0.279 *	0.076	0.001	−0.46	−0.10
	Patient	Physician	1.762 *	0.113	0.000	1.50	2.03
		Nurse	0.279 *	0.076	0.001	0.10	0.46
CAM therapies should be available to patients at the primary care level.	Physician	Nurse	−1.310 *	0.118	0.000	−1.59	−1.03
		Patient	−1.646 *	0.115	0.000	−1.92	−1.38
	Nurse	Physician	1.310 *	0.118	0.000	1.03	1.59
		Patient	−0.337 *	0.077	0.000	−0.52	−0.16
	Patient	Physician	1.646 *	0.115	0.000	1.38	1.92
		Nurse	0.337 *	0.077	0.000	0.16	0.52
CAM includes ideas and methods whose integration into the system of EBM can benefit everyone.	Physician	Nurse	−1.247 *	0.111	0.000	−1.51	−0.99
		Patient	−1.615 *	0.108	0.000	−1.87	−1.36
	Nurse	Physician	1.247 *	0.111	0.000	0.99	1.51
		Patient	−0.368 *	0.072	0.000	−0.54	−0.20
	Patient	Physician	1.615 *	0.108	0.000	1.36	1.87
		Nurse	0.368 *	0.072	0.000	0.20	0.54
Clinical medicine should integrate the belief-enhancing BM medicine.	Physician	Nurse	−1.320 *	0.112	0.000	−1.58	−1.06
		Patient	−1.517 *	0.109	0.000	−1.77	−1.26
	Nurse	Physician	1.320 *	0.112	0.000	1.06	1.58
		Patient	−0.197 *	0.073	0.018	−0.37	−0.03
	Patient	Physician	1.517 *	0.109	0.000	1.26	1.77
		Nurse	0.197 *	0.073	0.018	0.03	0.37
Healthcare professionals should be trained to talk to patients about the most commonly used CAM methods.	Physician	Nurse	−1.260 *	0.107	0.000	−1.51	−1.01
		Patient	−1.502 *	0.105	0.000	−1.75	−1.26
	Nurse	Physician	1.260 *	0.107	0.000	1.01	1.51
		Patient	−0.242 *	0.070	0.002	−0.41	−0.08
	Patient	Physician	1.502 *	0.105	0.000	1.26	1.75
		Nurse	0.242 *	0.070	0.002	0.08	0.41
Patients should consult their physician or therapist before using CAM.	Physician	Nurse	−0.514 *	0.101	0.000	−0.75	−0.28
		Patient	−0.625 *	0.099	0.000	−0.86	−0.39
	Nurse	Physician	0.514 *	0.101	0.000	0.28	0.75
		Patient	−0.110	0.066	0.216	−0.26	0.04
	Patient	Physician	0.625 *	0.099	0.000	0.39	0.86
		Nurse	0.110	0.066	0.216	−0.04	0.26
It’s a shame that CAM methods are not talked about enough and are rarely used.	Physician	Nurse	−1.446 *	0.123	0.000	−1.74	−1.16
		Patient	−1.544 *	0.120	0.000	−1.83	−1.26
	Nurse	Physician	1.446 *	0.123	0.000	1.16	1.74
		Patient	−0.098	0.080	0.442	−0.29	0.09
	Patient	Physician	1.544 *	0.120	0.000	1.26	1.83
		Nurse	0.098	0.080	0.442	−0.09	0.29
CAM methods are dangerous and harmful to health.	Physician	Nurse	1.371 *	0.104	0.000	1.13	1.62
		Patient	1.552 *	0.102	0.000	1.31	1.79
	Nurse	Physician	−1.371 *	0.104	0.000	−1.62	−1.13
		Patient	0.180 *	0.068	0.022	0.02	0.34
	Patient	Physician	−1.552 *	0.102	0.000	−1.79	−1.31
		Nurse	−0.180 *	0.068	0.022	−0.34	−0.02

*—the significance level value *p* < 0.05 indicates a statistically significant difference between the compared groups.

**Table 3:** Average values and standard deviation of respondents’ beliefs and attitudes about the effectiveness of CAM.

Statement	Physicians		Nurses		Patients	
	M	SD	M	SD	M	SD
Physical and mental health is maintained by internal energy or life force.	2.44	1.258	3.91	.999	3.94	0.802
Health and illness reflect the balance between life-enhancing and destructive forces.	2.44	1.282	3.71	1.043	3.76	0.830
The body heals itself, and the task of the healthcare professional is only to assist in the healing process.	1.99	1.010	3.06	1.121	3.22	1.035
The patient’s symptoms must indicate a general imbalance or dysfunction affecting the entire body.	2.48	1.096	3.61	0.998	3.79	0.781
The patient’s expectations, beliefs, and values must be integrated into the healthcare process.	3.80	0.791	3.95	0.970	3.97	0.796
Complementary and alternative methods are a threat to public health (R).	3.01	1.059	3.81	1.020	4.01	0.937
It makes me angry when people use CAM methods believing in their effectiveness (R).	3.13	0.906	3.76	1.090	3.62	0.994
Healthcare professionals (physicians, nurses, etc.) are embarrassed to talk about CAM with their colleagues.	2.73	0.874	2.84	1.049	2.91	0818
I am excited to think about the possibilities that may be hidden in CAM methods.	2.13	1.220	3.17	1.069	3.38	0.920
Therapies that have not been tested according to scientific principles must be banned.	1.86	1.110	3.15	1.051	2.96	0975
The effects of CAM therapies are most often the result of the placebo effect.	2.13	0.960	3.37	0.947	3.43	0.867
CAM therapies include ideas and methods from which EBM medicine can profit.	2.58	1.121	3.35	0.999	3.84	0.829
Most CAM therapies stimulate the body’s natural healing powers.	2.19	1.061	3.35	0.947	3.49	0.782
In the folk tradition and knowledge of our ancestors lie answers to questions in medicine that we do not know the answer to today.	2.06	1.062	3.38	1.012	3.34	0.784
The Divine/Higher Power possesses healing powers.	2.49	1.096	2.95	1.210	3.28	1.274

**Table 4:** Statistical significance of differences in beliefs and attitudes between respondents about the effectiveness of CAM: result of the Tukey test.

Statement	(i) By Profession/Status	(j) By Profession/ Status	Mean Value (ij)	Standard Error	*p* *	95% Confidence Interval	
						Lower Bound	Lower Bound
Physical and mental health is maintained by internal energy or life force.	Physician	Nurse	−1.473 *	0.108	0.000	−1.73	−1.22
		Patient	−1.504 *	0.105	0.000	−1.75	−1.26
	Nurse	Physician	1.473 *	0.108	0.000	1.22	1.73
		Patient	−0.031	0.070	0.897	−0.20	0.13
	Patient	Physician	1.504 *	0.105	0.000	1.26	1.75
		Nurse	0.031	0.070	0.897	−0.13	0.20
Health and illness reflect the balance between life-enhancing and destructive forces.	Physician	Nurse	−1.270 *	0.112	0.000	−1.53	−1.01
		Patient	−1.322 *	0.109	0.000	−1.58	−1.07
	Nurse	Physician	1.270 *	0.112	0.000	1.01	1.53
		Patient	−0.051	0.073	0.761	−0.22	0.12
	Patient	Physician	1.322 *	0.109	0.000	1.07	1.58
		Nurse	0.051	0.073	0.761	−0.12	0.22
The body heals itself, and the task of the healthcare professional is only to assist in the healing process.	Physician	Nurse	−1.069 *	0.122	0.000	−1.36	−0.78
		Patient	−1.231 *	0.119	0.000	−1.51	−0.95
	Nurse	Physician	1.069 *	0.122	0.000	0.78	1.36
		Patient	−0.162	0.079	0.103	−0.35	0.02
	Patient	Physician	1.231 *	0.119	0.000	0.95	1.51
		Nurse	0.162	0.079	0.103	−0.02	0.35
The patient’s symptoms must indicate a general imbalance or dysfunction affecting the entire body.	Physician	Nurse	−1.127 *	0.104	0.000	−1.37	−0.88
		Patient	−1.311 *	0.102	0.000	−1.55	−1.07
	Nurse	Physician	1.127 *	0.104	0.000	0.88	1.37
		Patient	−0.183 *	0.068	0.019	−0.34	−0.02
	Patient	Physician	1.311 *	0.102	0.000	1.07	1.55
		Nurse	0.183 *	0.068	0.019	0.02	0.34
The patient’s expectations. Beliefs and values must be integrated into the healthcare process.	Physician	Nurse	−0.150	0.099	0.285	−0.38	0.08
		Patient	−0.171	0.097	0.181	−0.40	0.06
	Nurse	Physician	0.150	0.099	0.285	−0.08	0.38
		Patient	−0.021	0.065	0.945	−0.17	0.13
	Patient	Physician	0.171	0.097	0.181	−0.06	0.40
		Nurse	0.021	0.065	0.945	−0.13	0.17
Complementary and alternative methods are a threat to public health (R).	Physician	Nurse	−0.803 *	0.113	0.000	−1.07	−0.54
		Patient	−1.005 *	0.110	0.000	−1.26	−0.75
	Nurse	Physician	0.803 *	0.113	0.000	0.54	1.07
		Patient	−0.202 *	0.073	0.017	−0.37	−0.03
	Patient	Physician	1.005 *	0.110	0.000	0.75	1.26
		Nurse	0.202 *	0.073	0.017	0.03	0.37
It makes me angry when people use CAM methods believing in their effectiveness (R).	Physician	Nurse	−0.633 *	0.117	0.000	−0.91	−0.36
		Patient	−0.486 *	0.114	0.000	−0.75	−0.22
	Nurse	Physician	0.633 *	0.117	0.000	0.36	0.91
		Patient	0.148	0.076	0.128	−0.03	0.33
	Patient	Physician	0.486 *	0.114	0.000	0.22	0.75
		Nurse	−0.148	0.076	0.128	−0.33	0.03
Healthcare professionals (physicians, nurses, etc.) are embarrassed to talk about CAM with their colleagues.	Physician	Nurse	−0.114	0.105	0.524	−0.36	0.13
		Patient	−0.178	0.103	0.195	−0.42	0.06
	Nurse	Physician	0.114	0.105	0.524	−0.13	0.36
		Patient	−0.063	0.069	0.626	−0.22	0.10
	Patient	Physician	0.178	0.103	0.195	−0.06	0.42
		Nurse	0.063	0.069	0.626	−0.10	0.22
I am excited about the possibilities hidden in CAM methods.	Physician	Nurse	−1.041 *	0.117	0.000	−1.32	−0.77
		Patient	−1.250 *	0.114	0.000	−1.52	−0.98
	Nurse	Physician	1.041 *	0.117	0.000	0.77	1.32
		Patient	−0.208 *	0.076	0.017	−0.39	−0.03
	Patient	Physician	1.250 *	0.114	0.000	0.98	1.52
		Nurse	0.208 *	0.076	0.017	0.03	0.39
Therapies that are not tested according to scientific principles must be banned (R).	Physician	Nurse	−1.293 *	0.117	0.000	−1.57	−1.02
		Patient	−1.101 *	0.114	0.000	−1.37	−0.83
	Nurse	Physician	1.293 *	0.117	0.000	1.02	1.57
		Patient	0.192 *	0.076	0.032	0.01	0.37
	Patient	Physician	1.101 *	0.114	0.000	0.83	1.37
		Nurse	−0.192 *	0.076	0.032	−0.37	−0.01
The effects of CAM therapies are most often due to the placebo effect (R).	Physician	Nurse	−1.244 *	0.104	0.000	−1.49	−1.00
		Patient	−1.296 *	0.102	0.000	−1.53	−1.06
	Nurse	Physician	1.244 *	0.104	0.000	1.00	1.49
		Patient	−0.052	0.068	0.724	−0.21	0.11
	Patient	Physician	1.296 *	0.102	0.000	1.06	1.53
		Nurse	0.052	0.068	0.724	−0.11	0.21
CAM therapies include ideas and methods that EBM medicine can learn from.	Physician	Nurse	−0.772 *	0.107	0.000	−1.02	−0.52
		Patient	−1.264 *	0.104	0.000	−1.51	−1.02
	Nurse	Physician	0.772 *	0.107	0.000	0.52	1.02
		Patient	−0.492 *	0.070	0.000	−0.66	−0.33
	Patient	Physician	1.264 *	0.104	0.000	1.02	1.51
		Nurse	0.492 *	0.070	0.000	0.33	0.66
Most CAM therapies stimulate the body’s natural healing powers.	Physician	Nurse	−1.162 *	0.101	0.000	−1.40	−0.92
		Patient	−1.301 *	0.099	0.000	−1.53	−1.07
	Nurse	Physician	1.162 *	0.101	0.000	0.92	1.40
		Patient	−0.139	0.066	0.087	−0.29	0.02
	Patient	Physician	1.301 *	0.099	0.000	1.07	1.53
		Nurse	0.139	0.066	0.087	−0.02	0.29
In the folk tradition and knowledge of our ancestors lie answers to questions in medicine that we do not know today.	Physician	Nurse	−1.320 *	0.105	0.000	−1.57	−1.07
		Patient	−1.281 *	0.102	0.000	−1.52	−1.04
	Nurse	Physician	1.320 *	0.105	0.000	1.07	1.57
		Patient	0.039	0.068	0.831	−0.12	0.20
	Patient	Physician	1.281 *	0.102	0.000	1.04	1.52
		Nurse	−0.039	0.068	0.831	−0.20	0.12
The Divine/Higher Power possesses healing powers.	Physician	Nurse	−0.463 *	0.141	0.003	−0.79	−0.13
		Patient	−0.792 *	0.137	0.000	−1.11	−0.47
	Nurse	Physician	0.463 *	0.141	0.003	0.13	0.79
		Patient	−0.329 *	0.092	0.001	−0.54	−0.11
	Patient	Physician	0.792 *	0.137	0.000	0.47	1.11
		Nurse	0.329 *	0.092	0.001	0.11	0.54

*—The significance level value *p* < 0.05 indicates a statistically significant difference between the compared groups.

## Data Availability

The original contributions presented in this study are included in the article/Supplementary Material. Further inquiries can be directed to the corresponding author.

## Supplementary Materials

The following supporting information can be downloaded at: https://www.mdpi.com/article/10.3390/healthcare13212818/s1.

## Abbreviations

CAM	Complementary and Alternative Medicine
CHBQ	CAM Health Belief Questionnaire
IMAQ	Integrative Medicine Attitude Questionnaire

## References

[1] National Center for Complementary and Integrative Health (NCCIH) (2016). Complementary, Alternative, or Integrative Health: What’s In a Name?. J. Altern. Complement. Med..

[2] World Health Organization (2013). WHO Traditional Medicine Strategy 2014–2023.

[3] Miroslav Krleža Lexicographic Institute (2021). Medicine. Croatian Encyclopedia. https://www.enciklopedija.hr/natuknica.aspx?id=2009.

[4] Mortada E.M. (2024). Evidence-Based Complementary and Alternative Medicine in Current Medical Practice. Cureus.

[5] Hoenders R., Ghelman R., Portella C., Simmons S., Locke A., Cramer H., Gallego-Perez D., Jong M. (2024). A review of the WHO strategy on traditional, complementary, and integrative medicine from the perspective of academic consortia for integrative medicine and health. Front. Med..

[6] Ashrafizadeh H., Rassouli M. (2023). Traditional, complementary, and alternative medicine in cancer care: Challenges and opportunities. Asia Pac. J. Oncol. Nurs..

[7] Zhang Y., Stokes J., Anselmi L., Bower P., Xu J. (2025). Can integrated care interventions strengthen primary care and improve outcomes for patients with chronic diseases? A systematic review and meta-analysis. Health Res. Policy Syst..

[8] Brosnan C., Dew K., Donovan S. (2023). Complementary and alternative medicine. Encyclopedia of Health Research in the Social Sciences.

[9] Josfeld L., Keinki C., Pammer C., Zomorodbakhsch B., Hübner J. (2021). Cancer patients’ perspective on shared decision-making and decision aids in oncology. J. Cancer Res. Clin. Oncol..

[10] Farooqui M., Hassali M.A., Abdul Shatar A.K., Shafie A.A., Seang T.B., Farooqui M.A. (2012). Complementary and Alternative Medicine (CAM) use by Malaysian oncology patients. Complement. Ther. Clin. Pract..

[11] Lee S.J. (2012). What does integrative medicine mean to the physician and the Ministry of Health and Welfare?. J. Korean Med. Assoc..

[12] Romero-García P.A., Ramirez-Perez S., Miguel-González J.J., Guzmán-Silahua S., Castañeda-Moreno J.A., Komninou S., Rodríguez-Lara S.Q. (2024). Complementary and alternative medicine (CAM) practices: A narrative review elucidating the impact on healthcare systems. Mechanisms and paediatric applications. Healthcare.

[13] Phang J.K., Kwan Y.H., Goh H., Tan V.I.C., Thumboo J., Østbye T., Fong W. (2018). Complementary and alternative medicine for rheumatic diseases: A systematic review of randomized controlled trials. Complement. Ther. Med..

[14] Kessel K.A., Klein E., Hack C.C., Combs S.E. (2018). Complementary medicine in radiation oncology: German health care professionals’ current qualifications and therapeutic methods. Strahlenther. Onkol..

[15] Kutschan S., Freuding M., Keinki C., Huebner J. (2020). Recommendations on complementary and alternative medicine within S3 guidelines in oncology: Systematic quality assessment of underlying methodology. J. Cancer Res. Clin. Oncol..

[16] Balneaves L.G., Watling C.Z., Hayward E.N., Ross B., Taylor-Brown J., Porcino A., Truant T.L. (2022). Addressing complementary and alternative medicine use among individuals with cancer: An integrative review and clinical practice guideline. J. Natl. Cancer Inst..

[17] Elwyn G., Durand M.A., Song J., Aarts J., Barr P.J., Berger Z., Cochran N., Frosch D., Galasiński D., Gulbrandsen P. (2017). A three-talk model for shared decision making: Multistage consultation process. BMJ.

[18] Chang K.H., Brodie R., Choong M.A., Sweeney K.J., Kerin M.J. (2011). Complementary and alternative medicine use in oncology: A questionnaire survey of patients and health care professionals. BMC Cancer.

[19] Carvalho V., Rangrej S.B., Rathore R. (2023). The Benefits of Integrative Medicine for Pain Management in Oncology: A Narrative Review of the Current Evidence. Cureus.

[20] Kocot-Kępska M., Zajączkowska R., Zhao J., Wordliczek J., Tomasik P.J., Przeklasa-Muszyńska A. (2021). The role of complementary and alternative methods in the treatment of pain in patients with cancer-current evidence and clinical practice: A narrative review. Contemp. Oncol..

[21] Hübner J., Münstedt K., Micke O., Prott F.J., Schmidt T., Büntzel J., Keinki C. (2023). Komplementäre oder alternative Medizin in der Onkologie: Chancen oder Risiken? [Complementary or alternative medicine in oncology: Chances or risks?]. Inn. Med..

[22] Sanford N.N., Sher D.J., Ahn C., Aizer A.A., Mahal B.A. (2019). Prevalence and nondisclosure of complementary and alternative medicine use in patients with cancer and cancer survivors in the United States. JAMA Oncol..

[23] Bahall M. (2017). Prevalence. Patterns and perceived value of complementary and alternative medicine among cancer 815 patients: A cross-sectional study. Descriptive study. BMC Complement. Altern. Med..

[24] Armano L., Vasiljev V., Rukavina T., Juraga D., Racz A., Tešić V. (2025). Bridging the gap: Attitudes and practices towards complementary and alternative medicine among oncology patients and healthcare professionals in Croatia. Front. Psychol..

[25] Loquai C., Dechent D., Garzarolli M., Kaatz M., Kaehler K.C., Kurschat P., Meiss F., Micke O., Muecke R., Muenstedt K. (2017). Use of complementary and alternative medicine: A multicenter cross-sectional study in 1089 melanoma patients. Eur. J. Cancer.

[26] Kwon J.H., Lee S.C., Lee M.A., Kim Y.J., Kang J.H., Kim J.Y., Lee H.J., Bae W.K., Kim M.J., Chie E.K. (2019). Behaviors and Attitudes toward the Use of Complementary and Alternative Medicine among Korean Cancer Patients. Cancer Res. Treat..

[27] Schils A., Lechon A.-S., Rondeaux S., Souard F., Van Laethem J.-L., Pochet S., Mathieu V., De Vriese C. (2023). Cancer patients’ behaviors and attitudes toward natural health products. BMC Complement. Med. Ther..

[28] Deng G. (2019). Integrative medicine therapies for pain management in cancer patients. Cancer J..

[29] Paolini B., Granetzke L., Wells R.E., Green M., Cowan R., Freitag F. (2019). Complementary and Alternative Approaches to Chronic Daily Headache: Part II—Manipulation-Based Therapies and Other CAM Therapies. 841 Chronic Headache.

[30] Marletta G., Canfora A., Roscani F., Cernicchiaro L., Cutrera M., Russo M., Artioli G., Sarli L. (2015). The complementary medicine (CAM) for treating chronic pain: Scientific evidence regarding the effects of healing touch massage. Acta Biomed..

[31] Genc E., Bulut I. (2024). Investigation of usage, attitudes toward complementary and alternative medicine among cancer patients in Turkey during the COVID-19 pandemic1. Work.

[32] Ozdelikara A., Karaoğlan İ.Ç. (2023). Effects of Oncology Patients’ Health Literacy on Use of Complementary and Alternative Therapy. Altern. Ther. Health Med..

[33] Koncz Z., Győrffy Z., Girasek E., Mátrai Z. (2022). Emlőrákban megbetegedett nők perioperatív viszonyulása a komplementer és alternatív medicinához az Országos Onkológiai Intézetben végzett felmérés alapján [Perioperative attitudes of breast cancer patients towards the complementary and alternative medicine based on a survey conducted at the National Institute of Oncology in Hungary]. Orvosi Hetil..

[34] Youn B.Y., Cha J.W., Cho S., Jeong S.M., Kim H.J., Ko S.G. (2023). Perception, attitudes, knowledge of using complementary and alternative medicine for cancer patients among healthcare professionals: A mixed-methods systematic review. Cancer Med..

[35] Alzahrani S.H., Bashawri J., Bakarman M.A. (2023). Attitudes and Practice of Health Care Professionals Toward Complementary and Alternative Medicine: A Cross-Sectional Study. BMC Complement. Med. Ther..

[36] Zhang Q., Peng W., Hou J., Wang Y., Dai Y. (2022). Integration of Complementary and Alternative Medicine into Mainstream Healthcare Systems: Current Evidence and Future Directions. Front. Public Health.

[37] Lie D., Boxer J. (2004). Development and validation of the CAM Health Belief Questionnaire (CHBQ) and CAM use and attitudes among medical students. BMC Med. Educ..

[38] Schneider C.D., Meek P.M., Bell I.R. (2003). Development and validation of IMAQ: Integrative Medicine Attitude Questionnaire. BMC Med. Educ..

[39] Bjerså K., Stener-Victorin E., Fagevik Olsén M. (2012). Knowledge about complementary. Alternative and integrative medicine (CAM) among registered health care providers in Swedish surgical care: A national survey among university hospitals. BMC Complement. Altern. Med..

[40] Sharp D., Lorenz A., Spring G., Little P., Hollinghurst S., Mercer S.W., MacPherson H. (2018). “Trying that way a square peg into a round hole”: A qualitative study of healthcare professionals’ views of integrating complementary medicine into primary care for musculoskeletal and mental health comorbidity. BMC Complement. Altern. Med..

[41] Zeliadt S.B., Douglas J.H., Gelman H., Coggeshall W., Taylor S.L., Kligler B., Bokhour B.G. (2022). Effectiveness of a whole health model emphasizing complementary and integrative health on reducing opioid use among patients with chronic pain. BMC Health Serv. Res..

[42] Shannon Z.K., Salsbury S.A., Gosselin D., Vining R.D. (2018). Stakeholders’ expectations from integrating chiropractic care into a rehabilitation setting: A qualitative study. BMC Complement. Altern. Med..

[43] Corina G., Christine H., Klein G. (2016). Oncologists’ experiences discussing complementary and alternative treatment options with their cancer patients: A qualitative analysis. Support. Care Cancer.

[44] Cutshall S., Derscheid D., Miers A.G., Ruegg S., Schroeder B.J., Tucker S., Wentworth L.M. (2010). Knowledge, attitudes. And use complementary and alternative therapies among clinical nurse specialists in the academic medical center. Clin. 902 Nurse Spec..

[45] DeKeyser F.G., Bar-Cohen B., Wagner N. (2001). Knowledge levels and attitudes of staff nurses in Israel towards complementary and alternative medicine. J. Adv. Nurs..

[46] Rojas-Cooley M.T., Grant M. (2009). Complementary and alternative medicine: Oncology nurses’ knowledge and attitudes. Oncol. Nurs. Forum..

[47] Samuels N., Zisk-Rony R.Y., Singer S.R., Dulitzky M., Mankuta D., Shuval J.T., Oberbaum M. (2010). Use of and attitudes towards complementary and alternative medicine among nurse-midwives in Israel. Am. J. Obstet. Gynecol..

[48] Risberg T., Kolstad A., Bremnes Y., Holte H., Wist E.A., Mella O., Klepp O., Wilsgaard T., Cassileth B. (2004). Knowledge of and attitudes towards complementary and alternative therapy: A national multicenter study of oncology professionals in Norway. Eur. J. Cancer.

[49] Thiago S.C.S., Tesser C.D. (2011). Family Health Strategy doctors’ and nurses’ perceptions of complementary therapies. Rev. Saúde Pública.

[50] Kreitzer M.J., Kligler B., Meeker W.C. (2009). Health professions education and integrative healthcare. Explore.

[51] van Vliet M., Jong M., Busch M., Meijer J.E.M., Von Rosenstiel I.A., Young M.C. (2015). Attitudes, Beliefs, and Practices of Integrative Medicine Among Nurses in the Netherlands. J. Holist. Nurs..

[52] Xu M., Yang C., Nian T., Tian C., Zhou L., Wu Y., Li Y., Deng X., Li X., Yang K. (2023). Adverse effects associated with acupuncture therapies: An evidence mapping from 535 systematic reviews. Chin. Med..

[53] Ventola C.L. (2010). Current issues regarding complementary and alternative medicine (CAM) in the United States: Part 1: The widespread use of CAM and the need for better-informed healthcare professionals to counsel patients. Phys. Ther..

[54] Block K.I., Block P.B., Gyllenhaal C. (2004). Integrative therapies in cancer: Modelling an evidence-based protocol. Integr. Cancer Ther..

[55] Sierpina V.S., Dalen I.S. (2010). The future of integrative medicine. Am. J. Med..

[56] Furlow M.L., Patel D.A., Sen A., Liu J.R. (2008). Physician and patient attitudes towards complementary and alternative medicine, obstetrics, and gynecology. BMC Complement. Altern. Med..

[57] Radovčić Z., Nola I.A. (2015). Integration of complementary and alternative medicine with primary health care in the Republic of Croatia—Opinions of primary health care physicians. Acta. Med. Croat..

[58] Jurković A., Racz A. (2020). Beliefs and attitudes of future health workers on the effectiveness of complementary and alternative medicine. J. Appl. Health Sci..

[59] Racz A., Crnković I., Brumini I. (2019). Nurses. Physiotherapists ‘ and sanitary engineers’ attitudes and beliefs about complementary and alternative medicine. Proceedings of the 6th International Multidisciplinary Scientific Conference SGEM SOCIAL SCIENCES & ARTS CONFERENCE.

[60] Zhao F.Y., Kennedy G.A., Cleary S., Conduit R., Zhang W.J., Fu Q.Q., Zheng Z. (2022). Knowledge about, attitude toward, and practice of complementary and alternative medicine among nursing students: A systematic review of cross-sectional studies. Front. Public Health.

[61] Siegel P., Broom A., Bowden V., Adams J., de Barros N.F. (2016). Attitudes towards complementary and alternative medicine among oncology professionals in Brazil. Complement. Ther. Med..

[62] Yang M.R., Zhang H., Ghana Z., Fan Y., Gu W., Ling C. (2018). Discrepancy in views of oncologists and cancer patients 9on complementary and alternative medicine in a Chinese general hospital. Integr. Cancer Ther..

[63] Zörgő S., Hernández O.L.O. (2018). Patient journeys of non-integration in Hungary: A qualitative study of possible reasons for considering medical modalities as mutually exclusive. Integr. Cancer Ther..

[64] Stratton T.D., McGivern-Snofsky J.L. (2008). Toward a sociological understanding of complementary and alternative medicine use. J. Altern. Complement. Med..

[65] Goldstein M.S. (2002). The emerging socio-economic and political support for alternative medicine in the United States. Ann. Am. Acad. Pol. Soc. Sci..

[66] Thomson P., Jones J., Browne M., Leslie S.J. (2014). Psychosocial factors that predict why people use complementary and alternative medicine and continue with its use: A population-based study. Complement. Ther. Clin. Pract..

[67] Nam J., Lee H., Lee S., Park H.J. (2024). Literature review of complementary and alternative therapies: Using text mining and analysis of trends in nursing research. BMC Nurs..

