# Supplementary material for: Comparing the Attitudes of Healthcare Professionals and Cancer Patients Toward the Integration and Perceived Effectiveness of Complementary and Alternative Medicine
Source: Healthcare (Basel). 2025 Nov 6;13(21):2818. doi: 10.3390/healthcare13212818 (PMC12609007; doi:10.3390/healthcare13212818)
Supplement: Supplementary file 1 [file healthcare-13-02818-s001.zip › healthcare-3906666-supplementary.pdf]

Supplementary Table S1. Level of agreement with statements on the integration of CAM into EBM medicine

Statement	Degree of agreement on a Likert scale of 1 – 5	Stratum					
		Patients		Health workers		Total	
		N	%	N	%	N	%
Although they don't discuss it, patients utilise some CAM therapies.	I completely disagree	10	2.4%	17	4.0%	27	3.2%
	I mostly disagree	31	7.6%	48	11.4%	79	9.5%
	Neither agree nor disagree	101	24.6%	139	32.9%	240	28.8%
	I mostly agree	181	44.1%	138	32.7%	319	38.3%
	I completely agree	87	21.2%	80	19.0%	167	20.1%
	Total	410	100.0%	422	100.0%	832	100.0 %
It is positive that there are complementary and/or alternative treatment methods	I completely disagree	10	2.4%	25	5.9%	35	4.2%
	I mostly disagree	21	5.1%	36	8.5%	57	6.9%
	Neither agree nor disagree	95	23.2%	149	35.3%	244	29.3%
	I mostly agree	186	45.4%	120	28.4%	306	36.8%
	I completely agree	98	23.9%	92	21.8%	190	22.8%
	Total	410	100.0%	422	100.0%	832	100.0 %
There is strong resistance among physicians to using CAM in patients involved in diagnostic and therapeutic processes.	I completely disagree	11	2.7%	10	2.4%	21	2.5%
	I mostly disagree	39	9.5%	53	12.6%	92	11.1%
	Neither agree nor disagree	194	47.3%	186	44.1%	380	45.7%
	I mostly agree	119	29.0%	131	31.0%	250	30.0%
	I completely agree	47	11.5%	42	10.0%	89	10.7%
	Total	410	100.0%	422	100.0%	832	100.0 %
A record of CAM use should be maintained in	I completely disagree	7	1.7%	13	3.1%	20	2.4%
	I mostly disagree	17	4.1%	30	7.1%	47	5.6%

the patient's medical record or documentation.	Neither agree nor disagree.	62	15.1%	87	20.6%	149	17.9%
	I mostly agree	197	48.0%	140	33.2%	337	40.5%
	I completely agree	127	31.0%	152	36.0%	279	33.5%
	Total	410	100.0%	422	100.0%	832	100.0 %
When taking an anamnesis, anamnestic data on CAM applications should be taken.	I completely disagree	5	1.2%	16	3.8%	21	2.5%
	I mostly disagree	11	2.7%	25	5.9%	36	4.3%
	Neither agree nor disagree	67	16.3%	76	18.0%	143	17.2%
	I mostly agree	205	50.0%	145	34.4%	350	42.1%
	I completely agree	122	29.8%	160	37.9%	282	33.9%
	Total	410	100.0%	422	100.0%	832	100.0 %
CAM should be integrated with EBM and conventional medicine methods.	I completely disagree	8	2.0%	28	6.6%	36	4.3%
	I mostly disagree	20	4.9%	62	14.7%	82	9.9%
	Neither agree nor disagree	71	17.3%	125	29.6%	196	23.6%
	I mostly agree	203	49.5%	117	27.7%	320	38.5%
	I completely agree	108	26.3%	90	21.3%	198	23.8%
	Total	410	100.0%	422	100.0%	832	100.0 %
The patient must inform their medical team of any CAM use.	I completely disagree	5	1.2%	9	2.1%	14	1.7%
	I mostly disagree	13	3.2%	16	3.8%	29	3.5%
	Neither agree nor disagree	49	12.0%	74	17.5%	123	14.8%
	I mostly agree	182	44.4%	137	32.5%	319	38.3%
	I completely agree	161	39.3%	186	44.1%	347	41.7%
	Total	410	100.0%	422	100.0%	832	100.0 %
The Croatian Health Insurance Fund (HZZO) should fully cover CAM treatment.	I completely disagree	14	3.4%	73	17.3%	87	10.5%
	I mostly disagree	15	3.7%	47	11.1%	62	7.5%
	Neither agree nor disagree	142	34.6%	158	37.4%	300	36.1%

	I mostly agree	163	39.8%	82	19.4%	245	29.4%
	I completely agree	76	18.5%	62	14.7%	138	16.6%
	Total	410	100.0%	422	100.0%	832	100.0 %
CAM therapies should be available to patients at the primary care level.	I completely disagree	13	3.2%	54	12.8%	67	8.1%
	I mostly disagree	17	4.1%	50	11.8%	67	8.1%
	Neither agree nor disagree	87	21.2%	142	33.6%	229	27.5%
	I mostly agree	187	45.6%	102	24.2%	289	34.7%
	I completely agree	106	25.9%	74	17.5%	180	21.6%
	Total	410	100.0%	422	100.0%	832	100.0 %
CAM encompasses ideas and methods that, when integrated into the system of EBM medicine, can benefit everyone.	I completely disagree	5	1.2%	35	8.3%	40	4.8%
	I mostly disagree	20	4.9%	51	12.1%	71	8.5%
	Neither agree nor disagree	50	12.2%	127	30.1%	177	21.3%
	I mostly agree	198	48.3%	124	29.4%	322	38.7%
	I completely agree	137	33.4%	85	20.1%	222	26.7%
	Total	410	100.0%	422	100.0%	832	100.0 %
Clinical medicine should integrate the best of CAM and EBM medicine.	I completely disagree	6	1.5%	32	7.6%	38	4.6%
	I mostly disagree	17	4.1%	31	7.3%	48	5.8%
	Neither agree nor disagree	43	10.5%	99	23.5%	142	17.1%
	I mostly agree	167	40.7%	136	32.2%	303	36.4%
	I completely agree	177	43.2%	124	29.4%	301	36.2%
	Total	410	100.0%	422	100.0%	832	100.0 %
Healthcare professionals should be trained to discuss the most commonly used complementary and alternative medicine	I completely disagree	4	1.0%	26	6.2%	30	3.6%
	I mostly disagree	11	2.7%	43	10.2%	54	6.5%
	Neither agree nor disagree	46	11.2%	105	24.9%	151	18.1%
	I mostly agree	189	46.1%	127	30.1%	316	38.0%
	I completely agree	160	39.0%	121	28.7%	281	33.8%

(CAM) methods with patients.	Total	410	100.0%	422	100.0%	832	100.0 %
Patients should consult their physician or a therapist before using CAM.	I completely disagree	4	1.0%	11	2.6%	15	1.8%
	I mostly disagree	9	2.2%	14	3.3%	23	2.8%
	Neither agree nor disagree	40	9.8%	83	19.7%	123	14.8%
	I mostly agree	169	41.2%	149	35.3%	318	38.2%
	I completely agree	188	45.9%	165	39.1%	353	42.4%
	Total	410	100.0%	422	therapists	832	100.0 %
It's a shame that CAM methods are rarely used or discussed enough.	I completely disagree	14	3.4%	50	11.8%	64	7.7%
	I mostly disagree	26	6.3%	50	11.8%	76	9.1%
	Neither agree nor disagree	110	26.8%	129	30.6%	239	28.7%
	I mostly agree	148	36.1%	96	22.7%	244	29.3%
	I completely agree	112	27.3%	97	23.0%	209	25.1%
	Total	410	100.0%	422	100.0%	832	100.0 %
CAM methods are dangerous and harmful to health.	I completely disagree	153	37.3%	115	27.3%	268	32.2%
	I mostly disagree	163	39.8%	114	27.0%	277	33.3%
	Neither agree nor disagree	85	20.7%	124	29.4%	209	25.1%
	I mostly agree	6	1.5%	49	11.6%	55	6.6%
	I completely agree	3	0.7%	20	4.7%	23	2.8%
	Total	410	100.0%	422	100.0%	832	100.0 %

Supplementary Table S2. Level of acceptance of claims about the effectiveness of CAM

Statement	Degree of agreement on a Likert scale 1 – 5	Stratum					
		Patients		Health workers		Total	
		N	%	N	%	N	%
Physical and mental health are maintained by internal energy or life force.	I completely disagree	1	0.2%	39	9.2%	40	4.8%
	I mostly disagree	12	2.9%	42	10.0%	54	6.5%
	Neither agree nor disagree	101	24.6%	94	22.3%	195	23.4%
	I mostly agree	190	46.3%	137	32.5%	327	39.3%
	I completely agree	106	25.9%	110	26.1%	216	26.0%
	Total	410	100.0%	422	100.0%	832	100.0%
Health and illness are a reflection of the balance between life- enhancing and destructive forces.	I completely disagree	3	0.7%	42	10.0%	45	5.4%
	I mostly disagree	17	4.1%	53	12.6%	70	8.4%
	Neither agree nor disagree	132	32.2%	105	24.9%	237	28.5%
	I mostly agree	180	43.9%	135	32.0%	315	37.9%
	I completely agree	78	19.0%	87	20.6%	165	19.8%
	Total	410	100.0%	422	100.0%	832	100.0%
The body heals itself, and the task of the healthcare professional is only to assist in the healing process.	I completely disagree	28	6.8%	73	17.3%	101	12.1%
	I mostly disagree	57	13.9%	90	21.3%	147	17.7%
	Neither agree nor disagree	164	40.0%	140	33.2%	304	36.5%
	I mostly agree	118	28.8%	84	19.9%	202	24.3%
	I completely agree	43	10.5%	35	8.3%	78	9.4%
	Total	410	100.0%	422	100.0%	832	100.0%
The patient's symptoms must be considered an indicator of a general imbalance or dysfunction affecting the entire body.	I completely disagree	4	1.0%	29	6.9%	33	4.0%
	I mostly disagree	9	2.2%	67	15.9%	76	9.1%
	Neither agree nor disagree	125	30.5%	124	29.4%	249	29.9%
	I mostly agree	202	49.3%	136	32.2%	338	40.6%
	I completely agree	70	17.1%	66	15.6%	136	16.3%
	Total	410	100.0%	422	100.0%	832	100.0%
The patient's expectations. Beliefs and values must be integrated into the healthcare process.	I completely disagree	3	0.7%	9	2.1%	12	1.4%
	I mostly disagree	8	2.0%	17	4.0%	25	3.0%
	Neither agree nor disagree	93	22.7%	98	23.2%	191	23.0%
	I mostly agree	199	48.5%	176	41.7%	375	45.1%
	I completely agree	107	26.1%	122	28.9%	229	27.5%
	Total	410	100.0%	422	100.0%	832	100.0%
Complementary and alternative methods are a threat to public health (R).	I completely disagree	6	1.5%	16	3.8%	22	2.6%
	I mostly disagree	18	4.4%	41	9.7%	59	7.1%
	Neither agree nor disagree	86	21.0%	138	32.7%	224	26.9%
	I mostly agree	153	37.3%	119	28.2%	272	32.7%
	I completely agree	147	35.9%	108	25.6%	255	30.6%
	Total	410	100.0%	422	100.0%	832	100.0%
It makes me angry when people use CAM methods	I completely disagree	10	2.4%	13	3.1%	23	2.8%
	I mostly disagree	18	4.4%	40	9.5%	58	7.0%

believing in their effectiveness (R).	Neither agree nor disagree	197	48.0%	162	38.4%	359	43.1%
	I mostly agree	79	19.3%	90	21.3%	169	20.3%
	I completely agree	106	25.9%	117	27.7%	223	26.8%
	Total	410	100.0%	422	100.0%	832	100.0%
Healthcare professionals (physicians, nurses, etc.) often feel embarrassed to discuss CAM with their colleagues.	I completely disagree	19	4.6%	51	12.1%	70	8.4%
	I mostly disagree	79	19.3%	86	20.4%	165	19.8%
	Neither agree nor disagree	255	62.2%	195	46.2%	450	54.1%
	I mostly agree	35	8.5%	69	16.4%	104	12.5%
	I completely agree	22	5.4%	21	5.0%	43	5.2%
	Total	410	100.0%	422	100.0%	832	100.0%
I am excited to think about the possibilities that may be hidden in CAM methods.	I completely disagree	16	3.9%	67	15.9%	83	10.0%
	I mostly disagree	37	9.0%	67	15.9%	104	12.5%
	Neither agree nor disagree	172	42.0%	166	39.3%	338	40.6%
	I mostly agree	145	35.4%	75	17.8%	220	26.4%
	I completely agree	40	9.8%	47	11.1%	87	10.5%
	Total	410	100.0%	422	100.0%	832	100.0%
Therapies that are not tested according to scientific principles must be banned (R).	I completely disagree	39	9.5%	75	17.8%	114	13.7%
	I mostly disagree	60	14.6%	70	16.6%	130	15.6%
	Neither agree nor disagree	215	52.4%	166	39.3%	381	45.8%
	I mostly agree	70	17.1%	67	15.9%	137	16.5%
	I completely agree	26	6.3%	44	10.4%	70	8.4%
	Total	410	100.0%	422	100.0%	832	100.0%
The effects of CAM therapies are most often the result of the placebo effect (R).	I completely disagree	10	2.4%	37	8.8%	47	5.6%
	I mostly disagree	35	8.5%	76	18.0%	111	13.3%
	Neither agree nor disagree	174	42.4%	172	40.8%	346	41.6%
	I mostly agree	152	37.1%	91	21.6%	243	29.2%
	I completely agree	39	9.5%	46	10.9%	85	10.2%
	Total	410	100.0%	422	100.0%	832	100.0%
CAM therapies include ideas and methods from which EBM medicine can profit.	I completely disagree	5	1.2%	36	8.5%	41	4.9%
	I mostly disagree	18	4.4%	55	13.0%	73	8.8%
	Neither agree nor disagree	93	22.7%	185	43.8%	278	33.4%
	I mostly agree	213	52.0%	94	22.3%	307	36.9%
	I completely agree	81	19.8%	52	12.3%	133	16.0%
	Total	410	100.0%	422	100.0%	832	100.0%
Most CAM therapies stimulate the body's natural healing powers.	I completely disagree	5	1.2%	43	10.2%	48	5.8%
	I mostly disagree	25	6.1%	65	15.4%	90	10.8%
	Neither agree nor disagree	177	43.2%	173	41.0%	350	42.1%
	I mostly agree	169	41.2%	99	23.5%	268	32.2%
	I completely agree	34	8.3%	42	10.0%	76	9.1%
	Total	410	100.0%	422	100.0%	832	100.0%
In the folk tradition and knowledge of our ancestors lie	I completely disagree	3	0.7%	56	13.3%	59	7.1%
	I mostly disagree	37	9.0%	53	12.6%	90	10.8%

the answers to questions in medicine that we do not know the answer to today.	Neither agree nor disagree	221	53.9%	172	40.8%	393	47.2%
	I mostly agree	115	28.0%	89	21.1%	204	24.5%
	I completely agree	34	8.3%	52	12.3%	86	10.3%
	Total	410	100.0%	422	100.0%	832	100.0%
The Divine/Higher Power possesses healing powers.	I completely disagree	58	14.1%	73	17.3%	131	15.7%
	I mostly disagree	41	10.0%	80	19.0%	121	14.5%
	Neither agree nor disagree	114	27.8%	150	35.5%	264	31.7%
	I mostly agree	121	29.5%	78	18.5%	199	23.9%
	I completely agree	76	18.5%	41	9.7%	117	14.1%
	Total	410	100.0%	422	100.0%	832	100.0%

Supp. 2 – Questionnaire

INFORMED CONSENT CAM

The informed consent form was drawn up by the Law on Health Care of the Republic of Croatia (Official Gazette 158/08, 71/10, 139/10, 22/11, 84/11, 12/12, 35/12, 70/12 and 82/13) and the Law on Patients' Rights of the Republic of Croatia (Official Gazette 169/04, 37/08). The research will ensure compliance with the Nuremberg Code and the Helsinki Declaration's fundamental ethical and bioethical principles (autonomy, justice, beneficence, and harmlessness).

Researcher: Ljerka Armano, MSc.Med.Tech., MSc.Biology. KBC Sestre milosrdnice, Vinogradska 29, Zagreb, mobile: 099 319 51 29, e-mail address: ljerka.armano@kbcsm.hr

Mentor/commentator: Assoc. Prof. prof. prim. Dr. PhD. Vanja Tesic, MD spec. epidemic; NHS Dr. Andrija Štampar; Assoc. Prof. Dr. Sc. Aleksandar Racz, Dr. Med. Spec.; University of Health Sciences, Zagreb

Title of the research: Beliefs and Attitudes about Complementary, alternative, and Integrative Medicine of Oncology Patients and Healthcare Workers in the Field of Oncology

Place research: Hospital Sisters charity, Hospital Zagreb

Source financing: The researcher alone finances this research, and nobody included will not receive any material or non-material compensation

Predicted duration research: Research to plan the spending of YOU and XII. monthly 2021.

Dear Sir/Madam,

We invite you to participate in a study exploring the attitudes and beliefs of healthcare professionals in oncology and oncology patients about alternative, complementary, and integrative medicine.

Please read this notice carefully. It will help you decide whether to participate in this scientific study. If you have any questions or concerns, please contact the researcher who provided you with this notice.

If you decide to participate in this research, you will be asked to sign this Informed Consent. The researcher signs the informed consent form, and a copy of the consent form can be obtained upon request. The researcher of this study uses the original informed consent form. An insight into the research results will be available to you after their publication, but even before that, you can get relevant information from the researcher about the research results.

ON research: According to WHO, complementary and alternative medicine (KAM) and integrative medicine supplement allopathic/conventional/western medicine. Complementary and alternative medicine (in further to the text KAM) per definition usually refers to a wide range of health care practices that are not part of a country's tradition and are not integrated into the dominant health care system (source: Traditional, Complementary and Integrative Medicine (who.int). Other terms besides alternative medicine that are sometimes used to describe these healthcare practices are natural medicine, paramedicine, unconventional medicine, and complete (holistic) medicine. Concrete examples of said medicine are Ayurvedic medicine, acupressure, acupuncture, chiropractic, bioenergetics, biomagnetic therapy, polarity therapy, feng shui, iridology, chelation therapy, chi kung, yoga, reiki, herbal medicine, etc.

Complementary or integrated medicine distinguishes alternative medicine from conventional medicine because it is used together with conventional medicine exclusively as a supplement, while alternative medicine is often used instead of conventional medicine. Despite the considerable growth of KAM and integrative medicine in Croatia and within the EU, there is still no single European classification of CAM.

However, the practice of CAM is widely present in Croatia and among patients, and the data is scarce. Research conducted among health workers shows the tendency to apply KAM among healthcare professionals. The research results could incentivize a critical review of the justification for integrating KAM into the Croatian healthcare system. At the same time, there is a lack of research attitudes health profession on KAM and integrative medicine, which is why the curricula of all health professions at undergraduate and graduate levels do not mention the contents at all, nor is there organized education at the level of lifelong learning, except education in acupuncture for doctors and specific massage techniques for physiotherapists. Therefore, a pilot study of beliefs and attitudes was designed as an exploratory, cross-sectional study at one point in time using the survey method using a specially designed questionnaire, on stratified, random, sample respondents (stratum 1 –

healthcare workers in oncology (doctors, nurses, physiotherapists, radiological technology engineers), 2 – oncology patients).

Research objectives:

The fundamental goal of the research is to determine the beliefs and attitudes of health workers and patients towards KAM and integrative medicine, focusing on several specific goals:

1. To determine differences in beliefs and attitudes about CAM and integrative medicine between individual strata (stratum 1 - healthcare professionals in oncology, substratum: physicians, nurses/technicians, and other healthcare professionals; stratum 2 - oncology patients).
2. Determine the prevalence and level of acceptance prejudice health workers and patients from oncological diseases and the widespread application of specific methods and techniques from the spectrum of KAM and integrative medicine.
3. To determine the connection between specific characteristics of the respondents (stratum 1 and 2) with expressed beliefs and attitudes about CAM and integrative medicine, especially the influence of several sociodemographic characteristics (e.g., age, basic education, place of work, worldview, financial status, etc.) as well as with regard to the type of morbidity they encounter in their professional work, personal experience with the use of CAM, personal experience of suffering from oncological diseases or the presence of such patients in the immediate family.
4. Determine the interest of healthcare workers in education in the field of CAM and integrative medicine, with the purpose of critical reconsideration, roundedness, and possible integrations of KAM in the education and healthcare system of the Republic of Croatia, and the attitudes of oncology patients about it.

Possible risks and benefits: There are no risks associated with this research. There is also no personal material gain from this research by anyone involved.

Right on refusal and withdrawal: Your participation in this research is voluntary. You can terminate your participation at any time without giving a reason.

Research permission: The research and informed consent were approved by the Ethics Committees of all institutions conducting research.

Confidentiality: Your personal and medical data will be collected according to ethical and

bioethical principles to ensure your privacy and protection of secrecy data. The researchers will not have access to the data in its original form. The data will be processed electronically, and you will be entered into the database using a code. Your name will never be revealed.

The research is conducted to create a doctoral dissertation, and the results will be used in purpose announcements, scientific works, and congress announcements, with the personal data of the respondents completely protected.

Contact information: For additional questions about the research itself, please contact the principal investigator:

Ljerka Armando, mag.med.techn.; Master of Science in Biology

Hospital Sisters charity, Vinogradska 29, Zagreb

Service for health care care, Department for Prevention and Prevention Hospital

Infection Tel.: 099 319 51 29

e-mail address: ljerka.armano@kbcsm.hr

By signing, I confirm that I have been informed about this research's objectives, benefits, and risks and agree to participate.

Place and date______________________

Participant's signature________________________

Signature research leader :

BELIEFS AND ATTITUDES ON COMPLEMENTARY, ALTERNATIVE AND INTEGRATIVE MEDICINE ONCOLOGICAL PATIENTS AND HEALTHCARE WORKERS IN THE ACTIVITY OF ONCOLOGY

Dear Sir/Madam,

Thank you for agreeing to participate in this scientific research. This questionnaire will take you approximately 10-15 minutes to complete. It is structured to collect information about attitudes and beliefs about complementary and alternative treatment methods (KAM) and the prevalence and types of application of CAM methods among healthcare professionals and patients.

The data collected in this research will be used exclusively to write a doctoral thesis at the Faculty of Medicine, University of Rijeka. The research is completely anonymous, and all responses will be analyzed at the group level.

Please answer everyone's questions more honestly. Also, if your question creates discomfort, it is your right not to respond to it.

It works simply. Questions are written in the male genus, and relationships equally apply to male and female genders. We thank you for your time, effort, and goodwill.

Leader research:

Ljerka Armando, mag. Med. Technical, M.Sc.Biol.

Clarification concepts:

According to the WHO, complementary and alternative medicine (CAM) and integrative medicine are supplements to conventional / Western medicine. Complementary and alternative medicine, according to the World Definition Health Organizations, indicate a broad series of practices protecting health that are not part of traditions in some countries and are not integrated into the dominant system. Other terms, besides alternative medicine, used to describe these healthcare practices are natural medicine, paramedicine, unconventional medicine, and complete (holistic) medicine. Concrete examples listed are Ayurvedic medicine, acupressure, acupuncture, chiropractic, bioenergetics, biomagnetic therapy, polarity therapy, feng shui, iridology, chelation therapy, chi kung, yoga, reiki, herbal medicine, etc.

Complementary or integrated medicine distinguishes alternative medicine from conventional medicine because it benefits together with conventional medicine exclusively as its supplement, while alternative medicine is used instead of conventional medicine.

I. DEMOGRAPHIC DATA ON RESPONDENT

1. Sex (round off)

a) male

b) female

2. Age (specify) completed years of life)

____

3. Conjugal status (round off)

a) married / in connection

b) unmarried/single

4. Level education (select highest finished degree education)

a) AXIS (basic school)

b) High school (medium professional qualifications)

c) University (Visa school preparation/bachelor's degree-180 ECTS)

d) University degree (high professional Bachelor's degree/Master's degree profession-300 ECTS)

5. Per occupation you are* (question to relations on healthcare workers):

a) doctor

b) medical nurse/technician

6. Specify the length of the working path for years* (question to relations on health workers)

____

7. Territory housing

a) urban middle (city)

b) rural middle (rural/suburban) territory)

8. Religious commitment

a) believer (believer practitioner)

b) agnostic (beige religious beliefs)

c) atheist (unbeliever)

9. Your income is:

a) below HR average

b) in height EN average (average net salary in the Republic of Croatia approx. 1,000 €)

c) above HR average

10. That whether you work in activities oncology* (question to relations only on healthcare workers)

a) that

b) not

II. ATTITUDES AND BELIEFS ON KAM

1. Integration of KAM with classical medicine

Please on a scale of 1 to 5, by choosing one number, indicate in to which measure you agree with the above claims

(1 – at all to not I agree, 2 – mainly to not I agree, 3 – nor I agree nor not I agree, 4 – I mostly agree, 5 – I agree)

Although on to that not they speak, patients in practice use some of therapy from areas KAM	1 2 3 4 5
Positively, there are complementary and alternative methods treatment	1 2 3 4 5
Among doctors, there is a powerful resistance application called KAM code patients who are included in diagnostic and therapeutic processes	1 2 3 4 5
Should have would lead records on application KAM in health cardboard/patient documentation	1 2 3 4 5
During taking anamnesis, one should take anamnestic data on applications KAM	1 2 3 4 5
WHERE should we integrate with methods classical, official medicine	1 2 3 4 5
Patient Sea informed its medical team about the application KAM	1 2 3 4 5
Treatment KAM should in be covered entirely through Croatian Health Insurance Institute	1 2 3 4 5

WHERE therapies should be available to patients on a level<br>primary health protection	1 2 3 4 5
WHERE includes ideas and methods that, by integration into rustling classical<br>medicine all I can to benefit	1 2 3 4 5
Clinical medicine should integrate the best of KAM and<br>of classical medicine	1 2 3 4 5
Health workers should be trained for conversations with<br>patients on most often applicable methods KAM	1 2 3 4 5
Patients would have to must ago applications to KAM to consult with yours<br>doctor or therapist	1 2 3 4 5
Damage is what to on WHERE methods too little speaks and what to rarely<br>use	1 2 3 4 5

2. Integration of KAM in educational and health system

Please on a scale of 1 to 5, by choosing one number, indicate in to which measure you agree with the above claims

(1 – at all to not I agree, 2 – mainly to not I agree, 3 – nor I agree nor not I agree, 4 – I mostly agree, 5 – I agree)

I know the difference between complementary and alternative medicine	1 2 3 4 5
Health employees should be educated in certain areas during<br>education through verified teaching plans and programs.<br>KAM	1 2 3 4 5
Health employees should have formal education from<br>areas KAM	1 2 3 4 5
During their formal education, health employees<br>they get very little or not at all information on KAM	1 2 3 4 5
Health employees do not they own enough knowledge<br>that patients can qualify to talk about possibilities<br>applications and effectiveness KAM	1 2 3 4 5
I would like to see healthcare professionals acquire enough knowledge through formal educational programs to be able to work with interested patients. could qualified to talk about possibilities applications and effectiveness KAM	1 2 3 4 5

Education from areas KAM should be an integral part educational plans and programs for all members of health professions	1 2 3 4 5
Education from KAM should be systematically integrated into various classical health contents (from anatomy to internal medicine and health care) on all levels of education, how theoretical so and practically	1 2 3 4 5
WHERE therapy, in principle, is dangerous for the patient and should is avoid	1 2 3 4 5
People who apply WHERE, and they are not health professionals they are ordinary charlatans and should would them prohibit work	1 2 3 4 5
It surprised me that people who do not understand that is the motive why therapist earnings and not patient welfare	1 2 3 4 5
WHERE therapists should pass rustling licensing as and health workers' classical medicine	1 2 3 4 5
Should have existed specialization from WHERE therapies	1 2 3 4 5
WHERE therapy should deal with only doctors	1 2 3 4 5
The state would determine who laughs to practice and provide KAM	1 2 3 4 5
I am proud of the health workers and patients who I can Open to talk on advantages and dangers use WHERE	1 2 3 4 5
I would when health workers had formal education from KAM	1 2 3 4 5

3. Belief in efficiency WHERE therapies

Please note that on a scale of 1 to 5, choose one number to indicate in which measure you agree with the above claims.

(1 – at all to not I agree, 2 – mainly to not I agree, 3 – nor I agree nor not I agree, 4 – I mostly agree, 5 – I agree)

Physical and mental health maintained is internal with energy or life by force	1 2 3 4 5
Health and disease reflection is a balance between life-empowering and destructive force	1 2 3 4 5

Body to self-healing and task is health worker only<br>help in the process of healing	1 2 3 4 5

Patients' symptoms must be considered indicators of general<br>imbalances or dysfunctions that involve the whole body	1 2 3 4 5
Patient's expectations, beliefs, and values must be<br>integrated in process health worries	1 2 3 4 5
Complementary and alternative methods threat are public to health	1 2 3 4 5
Angry me when people use WHERE methods believing in their efficiency	1 2 3 4 5
Health employees (doctors, nurses, etc.) they are ashamed of KAM<br>to talk with their colleagues	1 2 3 4 5
Exciting me is the thought of the possibility that maybe they hide in KAM<br>methods	1 2 3 4 5
Therapies that are not tested per scientific principles must be<br>forbidden	1 2 3 4 5
Effects WHERE therapy most often results in a placebo effect	1 2 3 4 5
WHERE therapies include ideas and methods of which classic medicine<br>can profit	1 2 3 4 5
Most WHERE therapy encourages natural healing strength in the body	1 2 3 4 5
IN national tradition and knowledge, our ancestors lie down answers to questions<br>in medicine to whom today, not we know the answer	1 2 3 4 5
I believe in Divine force and her healing to be able	1 2 3 4 5

4. How do you evaluate the possible therapeutic effectiveness of individual techniques WHERE?

Please indicate, by selecting a number, on a scale of 1 to 6, to what extent you assess the possible therapeutic effectiveness of each CAM technique.

(1 – completely ineffective, 2 – mainly ineffective, 3 – nor helps nor harm, 4 – primarily effective, 5 – completely effective, 6 – I have never heard of this technique)

Meditations	1 2 3 4 5 6
Massage techniques	1 2 3 4 5 6
Spiritual healings	1 2 3 4 5 6

Bioenergy	1 2 3 4 5 6
Prayer for yourself	1 2 3 4 5 6
Advocate prayer for another	1 2 3 4 5 6
Yoga	1 2 3 4 5 6

Medicinal plants	1 2 3 4 5 6
Chiropractic	1 2 3 4 5 6
Acupuncture	1 2 3 4 5 6
Homeopathy	1 2 3 4 5 6
Reiki	1 2 3 4 5 6
Hypnosis	1 2 3 4 5 6
Ayurveda	1 2 3 4 5 6
Osteopathy	1 2 3 4 5 6
Aromatherapy	1 2 3 4 5 6
Apitherapy	1 2 3 4 5 6
Naturopathy	1 2 3 4 5 6
Medical Marijuana	1 2 3 4 5 6

5. Reasons for applications KAM :

Please on a scale of 1 to 5, by choosing one number, indicate in to which measure you agree with the above claims.

(1 – at all to not I agree, 2 – mainly to not I agree, 3 – nor I agree nor not I agree, 4 – I mostly agree, 5 – I agree)

Dissatisfaction with Therapeutic Possibilities Classical medicine<br>(successful treatment outcomes, side effects of medications, helplessness in the terminal stages of the disease)	1 2 3 4 5
Dissatisfaction with the relationship between Doctors and Healthcare Workers Classical<br>medicine, according to the patient	1 2 3 4 5
Dissatisfaction with system health protection (lists waiting, expensiveness, and a lack of all medicines on lists that the HZZO covers.)	1 2 3 4 5
The belief that will WHERE help there where classic medicine more not	1 2 3 4 5

can help	
Fear of side effects of medicines and therapeutic procedures	1 2 3 4 5
The desire that the patient take over a more active role in their treatment	1 2 3 4 5
More extensive compliance WHERE method with personal life beliefs<br><br>and attitudes	1 2 3 4 5
Influence media and advertising	1 2 3 4 5
IN phase diseases patients they catch for each hope independently on<br><br>non-existence of evidence in efficiency	1 2 3 4 5
Repulsive me is the thought that they learn and apply methods that they are not<br><br>based on evidence	1 2 3 4 5

III. PERSONAL EXPERIENCE APPLICATIONS WHERE

1. Personally experience

Please on a scale of 1 to 5, by choosing one number, indicate in which measure you agree with the above claims (1 – at all to not I agree, 2 – mainly to not I agree, 3 – nor I agree nor not I agree, 4 – I mostly agree, 5 – I agree).

Personally alone, at least once in my life, I used some of the method<br><br>KAM.	1 2 3 4 5
Someone of members mine family or menu close people is at least<br><br>Once in life, some of the methods of KAM were used.	1 2 3 4 5
During treatment, met alone at least one patient who is<br><br>used some of the methods KAM.	1 2 3 4 5
Seriously I am thinking. To address to for help WHERE therapist.	1 2 3 4 5
I would very gladly use WHERE methods, but you are not. I can afford it from<br><br>financial reason	1 2 3 4 5

2. Have you, whether now or earlier, used any other preparations or methods for treatment for your illness other than what your oncologist and/or general practitioner prescribed?

a) YES – please specify what/which methods______

b) NOT

3. How often do you now take/practice WHERE?

a) never

b) rarely

c) periodically

d) once per week

e) each day

4. Reasons because of why you apply or would start applying WHERE

Please on a scale of 1 to 5, by choosing one number, indicate in which measure you agree with the above claims (1 – at all to not I agree, 2 – mainly to not I agree, 3 – nor I agree nor not I agree, 4 – I mostly agree, 5 – I agree).

If You do not use WHERE, please still answer all questions so that mark what. Why do you think certain patients use it?

Not I apply WHERE methods	1 2 3 4 5
Official medicine too much is toxic	1 2 3 4 5
Where close are my beliefs	1 2 3 4 5
I believe KAM	1 2 3 4 5
Imam feels that I hold control with their own hands	1 2 3 4 5
Official medicine too much is based on technology	1 2 3 4 5
Official medicine does not heal man as a whole	1 2 3 4 5
I want to try out all that can help	1 2 3 4 5
Disappointed alone because official medicine does not work	1 2 3 4 5
For prevention diseases	1 2 3 4 5
For treatment diseases	1 2 3 4 5
For the promotion of psychophysical health	1 2 3 4 5

5. Who are or would Your expectations of WHERE?

Please on a scale of 1 to 5, by choosing one number, indicate in which measure you agree with the above claims (1 – at all to not I agree, 2 – mainly to not I agree, 3 – nor I agree nor not I agree, 4 – I mostly agree, 5 – I agree).

If You do not use WHERE, please still answer all questions so that mark what. What do you think are the expectations from using CAM for those patients who use it?

Not that I use it, and I don't have expectations of KAM	1 2 3 4 5
That increase immunity	1 2 3 4 5
That helps in relaxation/sleeping	1 2 3 4 5
That helps in healing wound	1 2 3 4 5
That reduces the side effects of treatment	1 2 3 4 5
That improves the psychological/emotional state	1 2 3 4 5
That helps keep a good physical state	1 2 3 4 5

That directly affects/treats disease	1 2 3 4 5

6. **Is whether noticed some benefits WHERE?**

Please indicate on a scale of 1 to 5, by choosing one number, which measures you agree with the above claims (1—at all to not I agree, 2—mainly to not I agree, 3—neither I agree nor not I agree, 4 – I mostly agree, 5 – I completely agree).

If You do not use WHERE, please still answer all questions so that mark what. What do you think could be the benefits of using CAM for those patients who use it?

Relief hardship	1 2 3 4 5
Withdrawal diseases	1 2 3 4 5
High quality and quiet dream	1 2 3 4 5
Mitigation hurts	1 2 3 4 5
I didn't notice any benefits	1 2 3 4 5

7. **Is whether experienced some adverse/unwanted effects WHERE?**

Please on a scale of 1 to 5, by choosing one number, indicate in which measure you agree with the above claims (1 – at all to not I agree, 2 – mainly to not I agree, 3 – nor I agree nor not I agree, 4 – I mostly agree, 5 – I agree).

If you do not use CAM, please mark an X and answer all questions by indicating what you think could be the harmful or unwanted consequences of using CAM.

Digestive interference (e.g., diarrhea, vomiting, pains in the stomach...)	1 2 3 4 5
Disorder with blood pressure/pulse	1 2 3 4 5
Disorder sleep	1 2 3 4 5
Irritability	1 2 3 4 5
Difficult movement	1 2 3 4 5
Allergies	1 2 3 4 5
I didn't experience adverse effects	1 2 3 4 5
Health workers (doctors, pharmacists, nurses...) warned on possible harmfulness KAM	1 2 3 4 5

8. **If your doctor or medical sister cannot meet with you, you use WHERE? What prevents you from discussing this with them (or what do you think prevents patients)?**

Please on a scale of 1 to 5, by choosing one number, indicate in to which measure you agree with the above claims (1 – at all to not I agree, 2 – mainly to not I agree, 3 – nor I agree nor not I agree, 4 – I mostly agree, 5 – I agree).

If You do not use WHERE, please still answer all questions so that mark what. Why do you think CAM patients don't talk to their doctor or nurse about it?

Never me nobody it is not asked on to that	1 2 3 4 5
I am afraid. to that will me health employees ridicule	1 2 3 4 5
Health employees disapprove of WHERE	1 2 3 4 5
Health workers don't have enough knowledge about KAM	1 2 3 4 5
I am afraid. to that will me be denied/delayed treatment	1 2 3 4 5
Not that I feel too pleasant to talk about it	1 2 3 4 5
I consider that they don't need to inform	1 2 3 4 5
I tried alone, but they did not want to talk	1 2 3 4 5

1. **Of whom you have received information on WHERE?**

Please on a scale of 1 to 5, by choosing one number, indicate in to which measure you agree with the above claims (1 – at all to not I agree, 2 – mainly to not I agree, 3 – nor I agree nor not I agree, 4 – I mostly agree, 5 – I agree).

If you do not use WHERE, please still answer all questions so that you mark who you think patients get information about CAM from.

Health employees beyond hospitals	1 2 3 4 5
Health employees in the hospital	1 2 3 4 5
Friends or members of the family	1 2 3 4 5
Of other patients	1 2 3 4 5
Various associations (groups self-help)	1 2 3 4 5
TV, radio, Internet, magazines	1 2 3 4 5
IN to church	1 2 3 4 5

2. **Self-assessment health**

Please indicate on a scale of 1 to 5 how you rate your health by choosing one number.

(1 – very bad, 2 – bed, 3 – nor good, neither bad/mediocre, 4 – good, 5 – very good)

I consider that is my health	1 2 3 4 5

11. How much does WHERE burden Your budget?

Please indicate on a scale of 1 to 5, by choosing one number, which measures you agree with the above claims (1—at all to not I agree, 2—mainly to not I agree, 3—neither I agree nor not I agree, 4 – I mostly agree, 5 – I completely agree).

If you do not use KAM, please mark an X and still answer all the questions by indicating to what extent you think its application burdens the budget of those who do.

Not burdens or a little burdensome	1 2 3 4 5
It burdens or very burdensome	1 2 3 4 5
It is unimportant how much it costs because health is more important than money	1 2 3 4 5

12. Satisfaction services WHERE therapist

If you have used the services of a KAM therapist, please indicate on a scale of 1 to 5 to what extent you agree with the following statements by selecting a number.

(1 – at all to not I agree, 2 – mainly to not I agree, 3 – nor I agree nor not I agree, 4 – I mostly agree, 5 – I agree)

If YOU HAVE NOT used the services of a WHERE therapist, please Indicate that and do NOT ANSWER the following questions in the table below.

My experiences are positive and helped me am	1 2 3 4 5
Recommended I would WHERE to others	1 2 3 4 5
My experiences are positive, but I would not repeat	1 2 3 4 5
I didn't feel an improvement	1 2 3 4 5
My experience is negative	1 2 3 4 5
All is it deliberately works personally get a therapist	1 2 3 4 5
Departure WHERE to the therapist was my significant error	1 2 3 4 5

Thanks To you for your participation!

If you have questions, free to let me know by email at: ljerka.armano@kbcsm.hr
